# The interaction of RNA G-quadruplexes from the influenza A virus vRNA with TMPyP4 and BRACO-19 ligands

**DOI:** 10.1371/journal.pone.0335975

**Published:** 2025-11-19

**Authors:** Maria Nalewaj, Joanna Sliwiak, Karolina Zielinska, Pawel Zmora, Dorota Niedziałek, Grzegorz Wieczorek, Elzbieta Kierzek, Marta Szabat

**Affiliations:** 1 Institute of Bioorganic Chemistry, Polish Academy of Sciences, Noskowskiego, Poznan, Poland; 2 Ensemble3 sp. z o.o., Wólczyńska 133, 01-919 Warsaw, Warsaw, Poland; 3 Mossakowski Medical Research Institute, Polish Academy of Sciences, A. Pawińskiego 5, 02-106 Warsaw, Warsaw, Poland; NMIMS Deemed to be University - Mumbai Campus: NMIMS, INDIA

## Abstract

The influenza virus is an interesting research subject due to its serious threat to global public health. To date, various structural motifs from the influenza A virus (IAV) genome have been studied. Recently, RNA G-quadruplexes (G4s), noncanonical structures formed within the G-rich sequences of the IAV genome, have been reported. These motifs are suggested to be promising antiviral targets, and studying the G4 binding ligands has attracted increasing research interest. We hypothesized that RNA G4s can play a crucial role in IAV replication. This study focused on the interactions between RNA G4s and ligands, which have not been extensively studied in the influenza A virus California/4/2009 (H1N1) to date. Herein, commonly used G4-specific ligands, TMPyP4 and BRACO-19, were selected. First, we performed a reverse transcription stop assay to study the effect of both ligands on the inhibition of cDNA synthesis. Our results showed that both compounds inhibited this process in all wild-type G4 variants, with one variant exhibiting the most noticeable effect after the addition of TMPyP4. We also examined the binding affinity of TMPyP4 and BRACO-19 to IAV RNA G4s using isothermal titration calorimetry, circular dichroism, and fluorescence spectroscopy. Some differences in the binding properties of the three selected G4s were found. Furthermore, UV melting analysis was conducted to evaluate the effect of the ligands on the thermal stability of RNA G4s. To supplement our experimental approaches, we applied, in the limited range, molecular modeling to simulate the folding of 1Q G-quadruplex and provide further insights into its structural stability and topology. Finally, the influence of TMPyP4 on the IAV minireplicon activity was investigated, revealing significant inhibition of IAV replication. Overall, interactions between TMPyP4 and BRACO-19 with IAV G4s were demonstrated for the first time, suggesting that G4s can be potential anti-influenza drug targets.

## 1 Introduction

Influenza, commonly known as the flu, is a highly contagious disease that occurs seasonally as epidemics and occasionally as pandemics. The pandemic potential of the influenza virus is a result of its high genome variability and is correlated with its pathogenicity, replication, and growth kinetics. It is known that the secondary and tertiary structures of viral RNA control the above processes. Different RNA structural motifs are formed and used at the various stages of the viral replication cycle, highlighting their fundamental role in influenza virus biology.

Interestingly, the secondary structure of viral RNA (vRNA), including influenza A virus (IAV), is highly conserved across different viral strains, which suggests its importance during RNA replication. Furthermore, it has been shown that G-rich regions are present within the genomes of many viruses, for example, SARS-CoV-2 or HIV-1 [1,2]. These RNA regions can fold into noncanonical structures called G-quadruplexes (G4s) that are stabilized by Hoogsteen hydrogen bonds. It is assumed that they can have various important biological functions [2–5]. Therefore, understanding the vRNA structure-function relationship is essential, and research on this topic should be continued.

Recently, investigations concerning G-quadruplex binders as effective inhibitors of virus infections were published. For instance, Zou et al. used five G4 ligands, Ber, BRACO-19, 360A, PDS, and NiL, to study their interactions with the ZIKV RNA G4 structure [4]. The results revealed that PDS shows a high G4 binding affinity and thermal stability, as well as higher anti-ZIKV activity compared to the other G4 ligands.(4) Another report concerning G-quadruplexes as therapeutic targets was published by Lv and colleagues in 2022 [5]. The authors confirmed G4 structure formation within the chikungunya virus (CHIKV) genome using different biophysical techniques. Importantly, they demonstrated that BRACO-19 and TMPyP4 ligands inhibit CHIKV genome replication, showing that these compounds inhibit the production of infectious virions [5].

To date, a significant number of ligands binding G-quadruplexes have been studied [6–8]. Some are considered potential therapeutic agents [1,4,9]. Progress in the design and synthesis of new G4-interacting compounds allows for testing them as antiviral agents. Several such ligands have been identified and tested *in vitro* for their ability to inhibit viral replication [10–13]. Importantly, the biological relevance and *in vivo* therapeutic potential of these G4-targeting compounds remain areas of considerable interest.

Lately, we identified G-rich regions within the IAV A/California/4/2009 (H1N1) genome and examined their propensity to fold into G-quadruplexes using various biophysical methods [11]. Additionally, we showed that they are present within vRNA segments encoding polymerase complex proteins, indicating their possible role in the virus biology. Previously, Mergny and co-workers investigated the occurrence, localization, and variation of potential G-quadruplex-forming sequences in 77 H1N1 influenza genomes and provided a rational basis for targeting PQS motifs as a potential strategy against influenza [14].

In the current work, the primary goal was to investigate whether TMPyP4 and BRACO-19, well-characterized G4 ligands, could bind to the IAV RNA G-quadruplexes. Moreover, these compounds were used to study the differences in binding patterns of the selected G4-forming sequences and their mutated variants.

Here, we assessed whether G4-specific ligands influence the formation of vRNA G-quadruplexes using the reverse transcription stop assay. Next, we studied the interaction between the viral G4s and the selected ligands by isothermal titration calorimetry (ITC), circular dichroism (CD), and fluorescence spectroscopy. Additionally, the effect of ligand addition on the thermal stability of IAV RNA G4s was examined using UV melting analysis. To supplement our biophysical approaches, we applied, in the limited range, molecular modelling to simulate the folding of 1Q G-quadruplex and get more insights into its structural stability and topology. Finally, we investigated the effect of specific G4 ligands on virus replication using a minireplicon system.

To summarize, we report studies on the interaction between RNA G-quadruplexes from the IAV pandemic strain, A/California/4/2009, and TMPyP4 and BRACO-19 ligands. By analyzing the results obtained from various spectroscopic techniques, molecular biology methods, and biological assays, we demonstrated that selected G4-specific compounds differ in their binding properties. Moreover, our investigation confirms that G-quadruplex structures are formed within the influenza A virus genome and provides valuable data for the design of G4 stabilizers targeting viral genomes.

## 2 Materials and methods

### 2.1 Oligonucleotides and chemical compounds

Synthetic oligonucleotides were purchased from Genomed (Poland). Table 1 provides specific information about oligomer sequences and applications. The G4-specific ligands: TMPyP4 and BRACO-19 were bought from Sigma Aldrich (Germany).

**Table 1:** Characterization of selected G-rich sequences from the IAV genome.

Sequence name	Sequence (5′-3′)	Segment (Protein)	Segment length (nt)	Sequence location within segment
1Q	CUGGUGGGGCAGCAGCAAAGGGGAG	1 (PB2)	2341	436-460
7Q	GGUAGUGGUCCAUCAAUCGGGUUGAGCUGGGG	2 (PB1)	2341	2097-2128
11Q	GGAUGUAUAUUCUGAAAUGGGAGGCUGG	4 (HA)	1779	807-834

### 2.2 Reverse transcription stop assay

A reverse transcription stop assay was performed using SuperScript™ II Reverse Transcriptase (Thermo Fisher Scientific, USA). RNA oligonucleotides (2 pmol) and appropriate primers (2 pmol) were dissolved in a 10 mM potassium phosphate buffer (pH 6.8) containing 50 mM KCl and 0.1 mM EDTA to obtain a final volume of 10 µl. Samples were denatured at 85 °C for 5 minutes and then cooled down to room temperature overnight. After folding, the sample solutions were mixed with ligands (TMPyP4 or BRACO-19) at increasing concentrations (0, 6.25, 12.5, 25, 50, and 100 µM) and incubated at room temperature for 15 min. Next, the reaction mixture without RT enzyme was added to the sample solutions, gently mixed, and incubated at 42°C for 2 min. After that, the SuperScript™ II RT enzyme was added (200 units), mixed by pipetting up and down, and incubated at 42 °C for 50 min. The reaction was inactivated by heating at 70 °C for 15 min. The products of reactions were purified using the RNA Clean & Concentrator kit according to the manufacturer’s protocol (Zymo Research, Germany). Finally, electrophoresis of the products with the addition of 8 M urea was performed using 12% polyacrylamide denaturing gels, run at 140 V for 40 min in a cold room using the XCell SureLock Mini-Cell Electrophoresis System (Thermo Fisher Scientific, USA). The resultant gels were visualized using an Amersham Typhoon Biomolecular Imager and analyzed using ImageQuant™ TL 10.2 analysis software. IC_50_ values were calculated as the mean of three replicates using the free online tool, https://mythreyaherbal.com/easy-ic50-calculator-for-herbal-drug-and-bioassay-studies/. Graphs were created using OriginPro 2021 software.

### 2.3 UV melting analysis

The UV melting measurements were recorded on a Jasco V-650 spectrophotometer (Jasco Deutschland GmbH, Pfungstadt, Germany) equipped with a thermoprogrammer. Single-stranded oligonucleotide concentrations were calculated based on both the absorbance measured above 80 °C and the extinction coefficients, which were estimated by the nearest-neighbor model using the Integrated DNA Technologies calculator available on the website https://www.idtdna.com/calc/analyzer (Integrated DNA Technologies, Inc., Coralville, IA, USA). Samples (final concentration of 5 µM) were dissolved in the same buffer used for the RT stop assay and denatured at 90 °C for 5 min, then slowly cooled to room temperature. Next, the pre-folded RNAs were mixed with the tested ligands at a 1:1 ratio (final concentration of 5 µM) and incubated for 30 min at room temperature. Measurements for RNAs alone and in the presence of ligands were performed in triplicate using quartz cuvettes with a 0.1 cm path length and a sample volume of 30 µl. Absorbance versus temperature curves were obtained using the UV melting method at 260 nm and 295 nm wavelengths in a temperature range of 5–90 °C, with a heating rate of 0.2 °C/min. Thermal denaturation curves were analyzed, and the melting temperature (T_m_) values at 295 nm were determined as the mean of three replicates using OriginPro 2021 software. Graphs were also created using OriginPro 2021 software.

### 2.4 Circular dichroism spectroscopy

CD spectra were recorded on a Jasco J-815 spectropolarimeter (Jasco Deutschland GmbH, Pfungstadt, Germany) using 850 µl quartz cuvettes with a 5 mm path length and a sample volume of 600 µl. RNA oligonucleotides were dissolved in the same buffer as used for the RT stop assay to achieve a sample concentration of 6.8 nM. All samples were denatured for 5 min at 90 °C and then slowly cooled to room temperature overnight before data collection. The titration of the pre-folded RNA oligonucleotides was carried out by adding a solution of the tested ligands (TMPyP4 and BRACO-19) in 12 aliquots of 2.4 µl each, within a concentration range from 3.4 nM to 40.8 nM. Measurements were collected at 20 °C in the 210–350 nm wavelength range with a 1 nm data interval. CD measurements of RNA sequences from the PR8 genome were conducted in the same buffer as described above, at a final sample concentration of 5 µM, using 850 µl quartz cuvettes with a 5 mm path length and a sample volume of 600 µl. All samples were denatured at 90 °C for 5 min and then slowly cooled to room temperature overnight before data collection. Measurements were performed at 10 °C in the 220–340 nm wavelength range with a 1 nm data interval. All CD curves were established as an average of three CD measurements. The buffer spectrum was subtracted from the sample spectra. CD spectra were expressed as difference in molar absorption (Δɛ, in units of cm^2^ mmol^-1^) between right- and left-handed circularly polarized light and normalized for plotting and comparative purposes using the OriginPro 2021 software.

### 2.5 Fluorescence measurements

Fluorescence spectra were recorded using a JASCO J-815 CD/fluorescence spectropolarimeter (Jasco Deutschland GmbH, Pfungstadt, Germany) using 850 µl quartz cuvettes with a 5 mm path length and a sample volume of 600 µl. RNA oligonucleotides were dissolved in the same buffer used for the RT stop assay to achieve a final sample concentration of 6.8 nM. All samples were denatured for 5 min at 90 °C and then slowly cooled to room temperature overnight before data collection. The titration of the pre-folded RNA oligonucleotides was carried out by adding a solution of the tested ligands (TMPyP4 and BRACO-19) in 12 aliquots of 2.4 µl each, within a concentration range from 3.4 nM to 40.8 nM. Fluorescence spectra were measured in triplicate at 20 °C with a detector sensitivity of 900 V. For the TMPyP4 ligand, measurements were recorded from 600 to 800 nm with a 2 nm step, using an excitation wavelength of 433 nm. For the BRACO-19 ligand, from 500 to 650 nm with a 2 nm step, and using an excitation wavelength of 285 nm. Graphs were created using the OriginPro 2021 software.

### 2.6 Isothermal titration calorimetry (ITC)

Microcalorimetric measurements were carried out to yield thermodynamic insights into the interaction between IAV RNA G4s, their mutated variants, and two ligands (TMPyP4 and BRACO-19). The ITC experiments were conducted using MicroCal PEAQ-ITC equipment at 25°C in the same buffer as used for the RT stop assay. Titration of the ligand (kept at 350−1200 µM concentration in the syringe) against RNA 1Q, 7Q, and 11Q oligomers in the cell (kept at 8−30 µM concentration determined at 260 nm) was performed. Ligand solutions were injected in 19−38 aliquots of 2 µl until saturation was observed. Raw data were analyzed using MicroCal PEAQ-ITC Analysis Software with a One Set of Sites model (for interactions with BRACO-19) or Two Sets of Sites model (for interactions between TMPyP4 and wild-type of G4s) to obtain thermodynamic parameters, such as binding stoichiometry (N), dissociation constant (K_d_), and the changes in the enthalpy (ΔH) and entropy (ΔS). The ITC measurements were performed in duplicate. Data analysis and graphs were prepared using MicroCal PEAQ-ITC software, following the manufacturer’s instructions [15].

### 2.7 Molecular modelling of the 1Q G-quadruplex

The 1Q bimolecular G-quadruplex was constructed using an in-house RNA folding program, Nucleic Acids Bender, which operates based on tools and molecular dynamics implementation from the GROMACS-2025.2 molecular modelling suite [16]. Due to the strong restraints required during G-quadruplex folding, the preparation process for molecular dynamics simulations, aiming to replicate physiological conditions, involved a gradual reduction of restraint strength by approximately 35-fold concurrently with a gradual temperature increase from 0 to 310 K in the constant volume conditions (NVT). The resulting G-quadruplex was then subjected to multi-stage molecular dynamics simulations in constant pressure (NPT). Representative G-quadruplex geometries obtained every 10 ps from approximately 300 ns of the production runs of molecular dynamics simulation were clustered using the Jarvis-Patrick method with default parameters implemented in GROMACS into 22 distinct groups. The trajectories from all MD simulations were visually examined and analysed using VMD 1.9.4a57 software [17].

### 2.8 Cell line culture

The human embryonic kidney cell line HEK 293T was bought from LGC Standards, Poland. The cell line was maintained in high-glucose Dulbecco’s modified Eagle’s medium (DMEM; Thermo Fisher Scientific, USA) supplemented with 10% fetal bovine serum (FBS; Thermo Fisher Scientific, USA), 100 U/mL penicillin, and 100 μg/mL streptomycin. The cells were incubated at 37 °C in a humidified incubator with 5% CO_2_.

### 2.9 Luminescent cell viability assay

The cytotoxicity of ligands was tested using Cell Titter-Glo® cell viability reagent according to the manufacturer’s protocol (Promega, USA). Briefly, HEK 293T cells were seeded at 1 x 10^5^ cells/ml in 96-well plates, along with the tested compounds (TMPyP4, TMPyP2, and BRACO-19 ligands). The cells were grown for 48 hours. Cell Titter-Glo® reagent was added to the cell supernatant in a 96-well plate, and after 2 minutes of mixing and 10 minutes of incubation, the solution was transferred to white plates. Finally, the luminescence measurements were conducted using the HIDEX microplate reader.

### 2.10 Preparation of IAV plasmids and the minireplicon construct

The minireplicon system used in this study consists of pCAGGS (A/IvPR/8/34) plasmids encoding the PB1, PB2, PA, and NP proteins of the influenza A virus (strain A/Puerto Rico/8/1934 H1N1) and the pPolI-LucRT plasmid encoding the firefly luciferase gene under the control of the influenza A segment 8 5´-end promoter (a kind gift from Prof. Stefan Poehlmann, German Primate Center – Leibniz Institute for Primate Research, Goettingen, Germany). To generate the California 2009 minireplicon, the protein-coding regions of pCAGGS plasmids (A/IvPR/8/34) for the PR8 strain were replaced with sequences encoding the same proteins from the influenza A virus strain A/California/07/2009(H1N1). The cloning method used to create these plasmids is called Gibson assembly. For this, we designed primers (see Table 1) in the SnapGene program and used GeneArt™ Gibson Assembly HiFi Master Mix (Invitrogen) for plasmid preparation. The procedure was carried out according to the manufacturer’s protocol with minor modifications.

### 2.11 IAV minireplicon assay

This assay was performed as previously described with minor modifications.(18) HEK 293T cells were seeded in a 24-well plate at a density of 4 x 10^5^ cells/ml per well and incubated overnight at 37 °C. Cultured cells were co-transfected with pCAGGS plasmids prepared as described above at various concentrations (California 2009: 150 ng of PB1, PB2, PA, and Luc, and 300 ng of NP; Puerto Rico 8: 50 ng of PB1, PB2, PA, and Luc, and 100 ng of NP) using Lipofectamine™ 2000 reagent at 80% cell confluency according to the manufacturer’s protocol (Thermo Fisher Scientific, USA). Plasmids containing GFP (1 µg) or pCMV-Luc Firefly Luciferase mammalian expression reporter vector (pCMV-Luc, 200 ng) (Origene) served as control probes. Next, the TMPyP4 or TMPyP2 compounds at the final concentrations (1.25, 3.125, 6.25 or 3.125, 6.25, 12.5 µM, respectively) were added to the co-transfected cells. After 18 hours of incubation at 37 °C, the cell medium was replaced with fresh DMEM medium containing FBS and antibiotics, and the cells were incubated for an additional 30 hours at 37 °C. Next, the cells were collected into Eppendorf tubes and centrifuged at 3000 rpm for 3 minutes. Then, the cell pellet was resuspended in 250 µl of ONE-Glo™ luciferase reagent (Promega, USA) and incubated at room temperature for 15 minutes to ensure complete cell lysis. Finally, 50 µl of each sample lysate was transferred in triplicate to a 96-well white polystyrene plate, and the luminescence was measured using a HIDEX microplate reader. Mean luciferase expression and standard deviations (SDs) were calculated from luminescence measurements of six separate wells. The data analysis was performed using OriginPro 2021 and GraphPad software.

## 3 Results

Our previous studies identified 12 potential quadruplex-forming sequences (PQSs) within the IAV A/California/4/2009 (H1N1) genome and determined their propensity to fold into RNA G-quadruplex structures [11]. By combining bioinformatics tools and biophysical methods, we found that three PQS motifs are located within the PB1, PB2, and HA genome segments and can form stable G4s. During the influenza virus replication cycle, complementary RNA (cRNA) is synthesized from vRNA by the RNA-dependent RNA polymerase (RdRp). Additionally, in the cell nucleus, vRNA is transcribed into mRNA, which serves as a template for the translation of viral protein. Therefore, the presence of selected G-quadruplexes within the vRNA encouraged us to investigate their potential role during the viral replication cycle. We hypothesize that G-quadruplex structures form within the IAV genome and can be implicated in viral replication regulation. In this study, we selected three PQS motifs that have been previously validated to adopt G4 structures using various biophysical methods [19]. We assumed that the sequences 1Q, 7Q, and 11Q have the highest propensity to fold into G4s.

**Fig 1:**
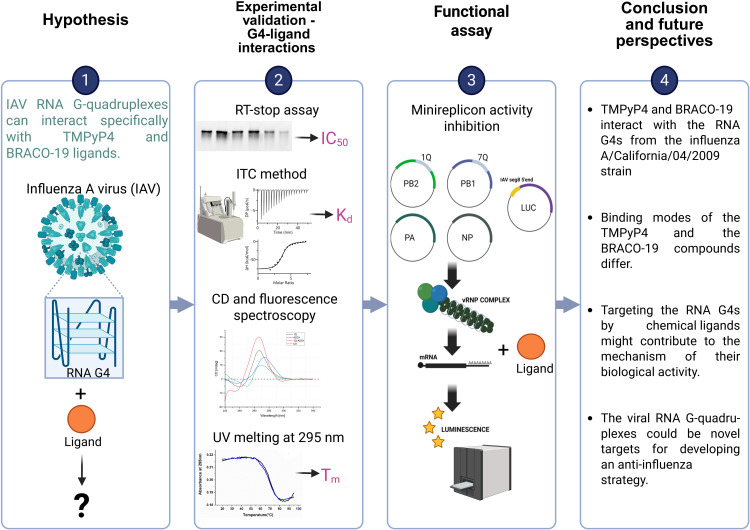
Scheme of the experimental design and workflow of the study.

Herein, we investigated the formation and structural features of IAV RNA G-quadruplexes upon the addition of G4-specific ligands using various biophysical techniques, including RT stop assay, ITC technique, CD, UV melting, and fluorescence spectroscopy. Additionally, in the limited range, we examined the influence of the TMPyP4 compound on viral replication within the IAV minireplicon system. Fig 1 illustrates the experimental workflow used in our study. The characteristics of G-rich sequences are summarized in Table 1, whereas the oligomer sequences used in this study, along with their applications, are presented in S1 Table (Supplementary Materials).

### 3.1 G4-specific ligands inhibit the proceeding of reverse transcription by interacting with G4-forming sequences

Overall, a native polyacrylamide gel electrophoresis (PAGE) can provide valuable information about the binding affinity of ligands to G-quadruplex structures and their conformational preferences. Therefore, given the findings that G-quadruplexes can induce reverse transcription pausing, [12,13] we employed an RNA-dependent DNA polymerase stop assay, called a reverse transcription (RT) stop assay. In this experiment, we used denaturing polyacrylamide gel electrophoresis to more accurately assess the quantity of the product after the RT reaction.

In the RT stop assay, the enzyme proceeds along the RNA template until it encounters a stable, ligand-interacting RNA G4 structure. Therefore, we assumed that the cDNA synthesis catalyzed by reverse transcriptase would be inhibited after adding a G4-specific ligand to the reaction. Moreover, we postulated that the synthesis of the full-length product would be more significantly arrested with an increasing ligand concentration. However, in the case of G4 variants mutated within the G-rich regions, inhibition of cDNA synthesis was expected to be less noticeable during PAGE analysis. Importantly, before the reverse transcription reaction, all RNA templates were folded in a buffer containing potassium cations required for stable G-quadruplex formation. Then, the G4 variants were incubated with G4-specific chemical compounds, ensuring ligand binding to the structure. In our experiments, we used two commercially available ligands, TMPyP4 and BRACO-19. Additionally, the TMPyP4 analog, TMPyP2, which has a known lower affinity to G-quadruplex structures, was used as a control [18–21].

**Fig 2:**
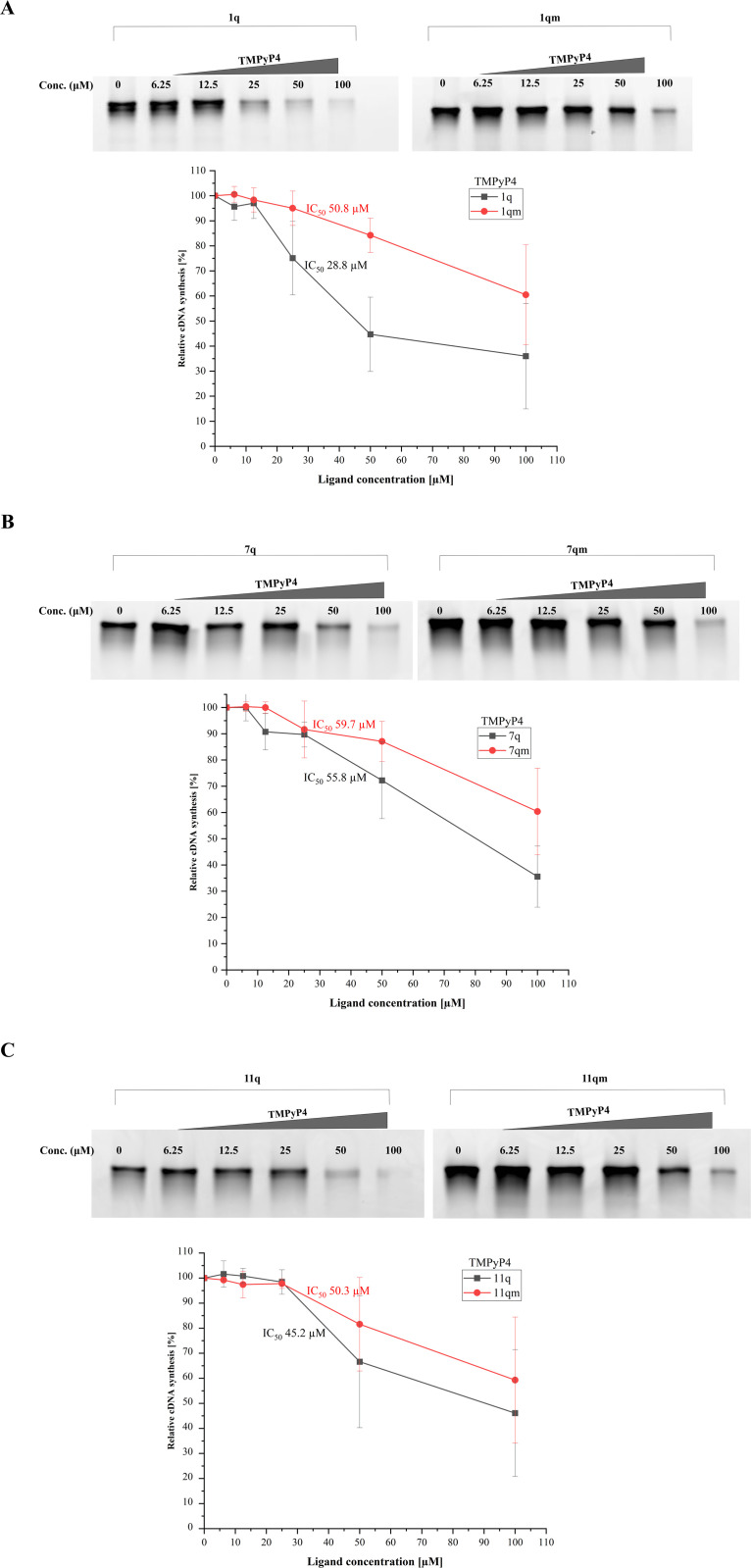
The results of the inhibition of cDNA synthesis catalyzed by the reverse transcriptase in the presence of TMPyP4; the resultant gels (upper) and the plots of band intensity vs. ligand concentration (bottom) for 1q/1qm, 7q/7qm, and 11q/11qm variants (panel A, panel B, and panel C, respectively). The most representative gel results for each sequence are presented here. The IC_50_ values were obtained as the average of three replicates.

**Fig 3:**
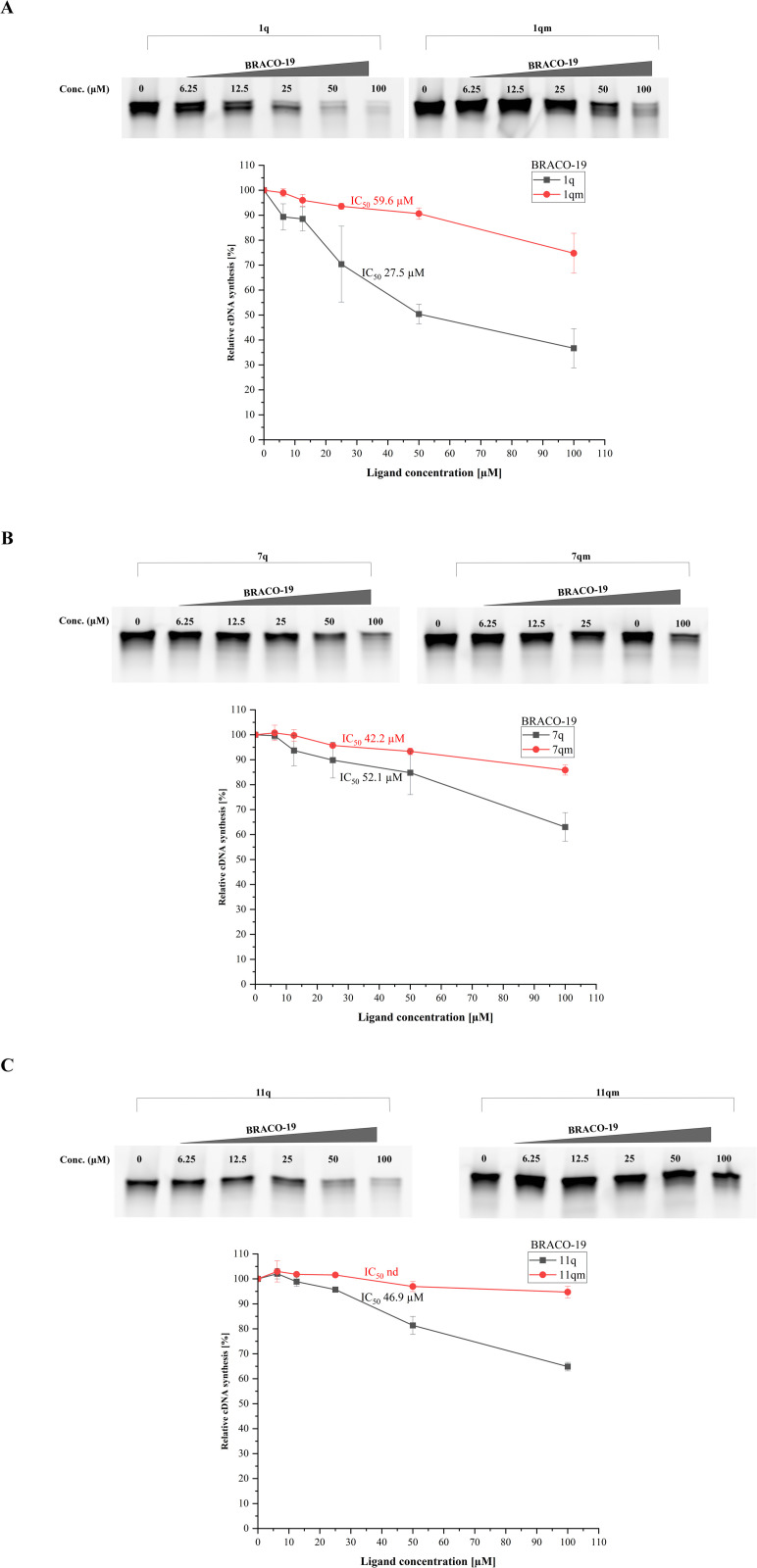
The results of the inhibition of cDNA synthesis catalyzed by the reverse transcriptase in the presence of BRACO-19; the resultant gels (upper) and the plots of band intensity vs. ligand concentration (bottom) for 1q/1qm, 7q/7qm, and 11q/11qm variants (panel A, panel B, and panel C, respectively). The most representative gel results for each sequence are presented here. The IC_50_ **values were obtained as the average of three replicates.**

Based on our results, we reported that the reverse transcription process was hindered by both G4-specific compounds for all wild-type G4 variants (1q, 7q, and 11q). We provided representative gels for three RNA G-quadruplexes with TMPyP4 and BRACO-19 ligands (Figs 2 and 3, respectively). To quantify the effect of ligands on the RT reaction, the IC_50_ values were determined as the average of three replicates. The IC_50_, the half-maximal inhibitory concentration, is a commonly used parameter to compare drug/ligand potency. The IC_50_ values are presented in Figs 2 and 3. Because the products of RT stop reactions were cleaned as described in the Materials and Methods section, the resultant gels display only the full-length products. The full PAGE images are shown in the Supplementary Materials (S1 and S2 Figs). Moreover, the gels obtained for the G4s with the TMPyP2 compound are presented in Supplementary Materials (S3 Fig).

Overall, the gel analysis revealed some differences in the reverse transcription pausing between wild-type and mutant sequences. According to the resulting gels in Fig 2, cDNA synthesis catalyzed by SuperScript II reverse transcriptase was inhibited in all cases (1q, 7q, and 11q) after the addition of TMPyP4 ligand. We assume that ligand binding to the sequence promotes the formation of a G4 structure. Therefore, there is no significant difference in the band intensities between wild-type and mutated variants without the ligand addition. As expected, we observed that the full-length product synthesis was more significantly inhibited in the wild-type G4s compared to the mutated variants with increasing TMPyP4 concentration (Fig 2). Interestingly, when comparing the results for 1q/1qm versus 7q/7qm, remarkable differences in the level of RT inhibition were displayed after the ligand addition. Specifically, a significant change in band intensity between 1q and 7q variants is already noticeable at 25 µM TMPyP4 concentration (Fig 2A vs. 2B). Similar effects were noted for 11q and 7q, as indicated by the gel electrophoresis patterns in Fig 2B and 2C. On the other hand, product synthesis inhibition observed after 100 µM TMPyP4 addition (also to mutated variants) indicates the nonspecific reaction inhibition unrelated to G-quadruplex structure formation. This suggests that such a TMPyP4 concentration may be too high to be used as evidence of G4 structure-ligand interaction. Furthermore, we revealed different band patterns for the tested compounds by comparing the obtained results for TMPyP4 (Fig 2) and TMPyP2 (S3 Fig). The significant differences in band intensity between wild-type (1q, 7q, and 11q) and mutated variants (1qm, 7qm, and 11qm) with increasing TMPyP2 concentration were not found (S3 Fig). This confirms that this compound is less specific toward RNA G-quadruplex structures.

Our analysis of the IC_50_ parameter shows that the 1q variant with TMPyP4 ligand has the lowest value (28.8 µM, Fig 2A), whereas 7q and 11q have higher values of 55.8 µM and 45.2 µM, respectively (Fig 2B and C, respectively). This suggests that the TMPyP4 ligand can bind more tightly to the 1q motif than to 7q and 11q. Additionally, we noticed that the IC_50_ values calculated for mutated variants, 1qm and 7qm (50.8 µM and 59.7 µM, respectively, Fig 2A and B), are much higher than those for wild-type motifs. This observation may indicate the specificity of the interaction between RNA G4s and the TMPyP4 compound. However, a different effect was seen for 11q compared to 11qm, which could be due to differences in band intensity on the gel (Fig 2C). Furthermore, it is worth mentioning that in a few cases, slightly higher band intensities compared to the control (RNA template without the ligand) may result from differences in product concentration after the purification on the columns. The RT stop assay results indicate the stabilization of G4 after the ligand addition. Moreover, the different patterns of intensity on the gels may result from different stabilities of the RNA G-quadruplexes.

Considering the RT stop assay with the BRACO-19 compound, the resulting gels showed a slightly different band pattern (Fig 3) than after adding TMPyP4. We observed that BRACO-19 can influence RT processing; however, the effect appeared less noticeable based on the gel analyses. Nevertheless, the band intensity for 1q differs from the 1qm variant (Fig 3A), indicating more effective reverse transcription inhibition by BRACO-19 for the wild-type variant (1q) (Fig 3A). Moreover, we noticed less remarkable changes in band intensity for the 7q/7qm variants (Fig 3B). In the case of 11q G4, a similar effect to that observed for 1q G4 was noted, with a noticeable change appearing between 25 and 50 µM BRACO-19 concentration (Fig 3C).

As in the case of TMPyP4, we determined the IC_50_ values for three RNA G4s and the BRACO-19 compound, as shown in Fig 3. Based on the results, the lowest IC_50_ value was obtained for the 1q variant (27.5 µM, Fig 3A). On the other hand, we found that BRACO-19 can bind to 7q and 11q with similar affinity (IC_50_ values = 52.1 µM and 46.9 µM, respectively, Fig 3B and C). We suggest that the BRACO-19 ligand is characterized by a higher binding affinity to RNA G-quadruplexes than the TMPyP4 compound, as indicated by its lower IC_50_ value.

Overall, the RT stop assay results suggest that RNA sequences capable of G4 formation are stabilized after the addition of tested ligands (TMPyP4 and BRACO-19). However, it is worth noting that the above compounds are not very active. The enzyme-pausing effect caused by these ligands required relatively high concentrations (Figs 2 and 3), which may indicate low selectivity of the selected compounds for IAV RNA G4 structures or moderate stabilization of the G-quadruplexes. The TMPyP2 analog did not affect cDNA synthesis (resulting gels shown in S3 Fig in Supplementary Materials). Based on this observation, we can conclude that two chemical compounds, TMPyP4 and BRACO-19, can interact with the IAV RNA G-quadruplexes; however, TMPyP2 has a low affinity toward the G4s.

In summary, reverse transcription of the RNA G4-containing sequence revealed a clear pausing site corresponding to the predicted G4 motif. The addition of a G4-specific ligand caused a dose-dependent increase in RT stalling. Moreover, control experiments using a mutated (non-G4-forming) sequence showed less significant pausing, confirming the specificity of the interaction. However, the differences observed in the gel analysis may result from several reasons, such as G4 structure formation, folding topology, molecularity, or the mechanism of ligand binding. Additionally, methodological and assay-related factors may influence the different band patterns and signal intensities observed on the gels.

### 3.2 Characterization of the interaction between the IAV RNA G-quadruplexes and ligands by isothermal titration calorimetry (ITC)

Isothermal titration calorimetry (ITC) is a method used to determine thermodynamic features of biomolecular interactions. It has been proven useful in studying the folding kinetics, ligand binding specificity, and energetic aspects of G-quadruplex structure-ligand interactions [4,22,23]. Several examples of ITC applications in studies of ligand binding properties to G4s have been reported [22–24]. Hence, our investigations employed the ITC method to examine the interactions between TMPyP4 and BRACO-19 with IAV RNA G4s.

**Fig 4:**
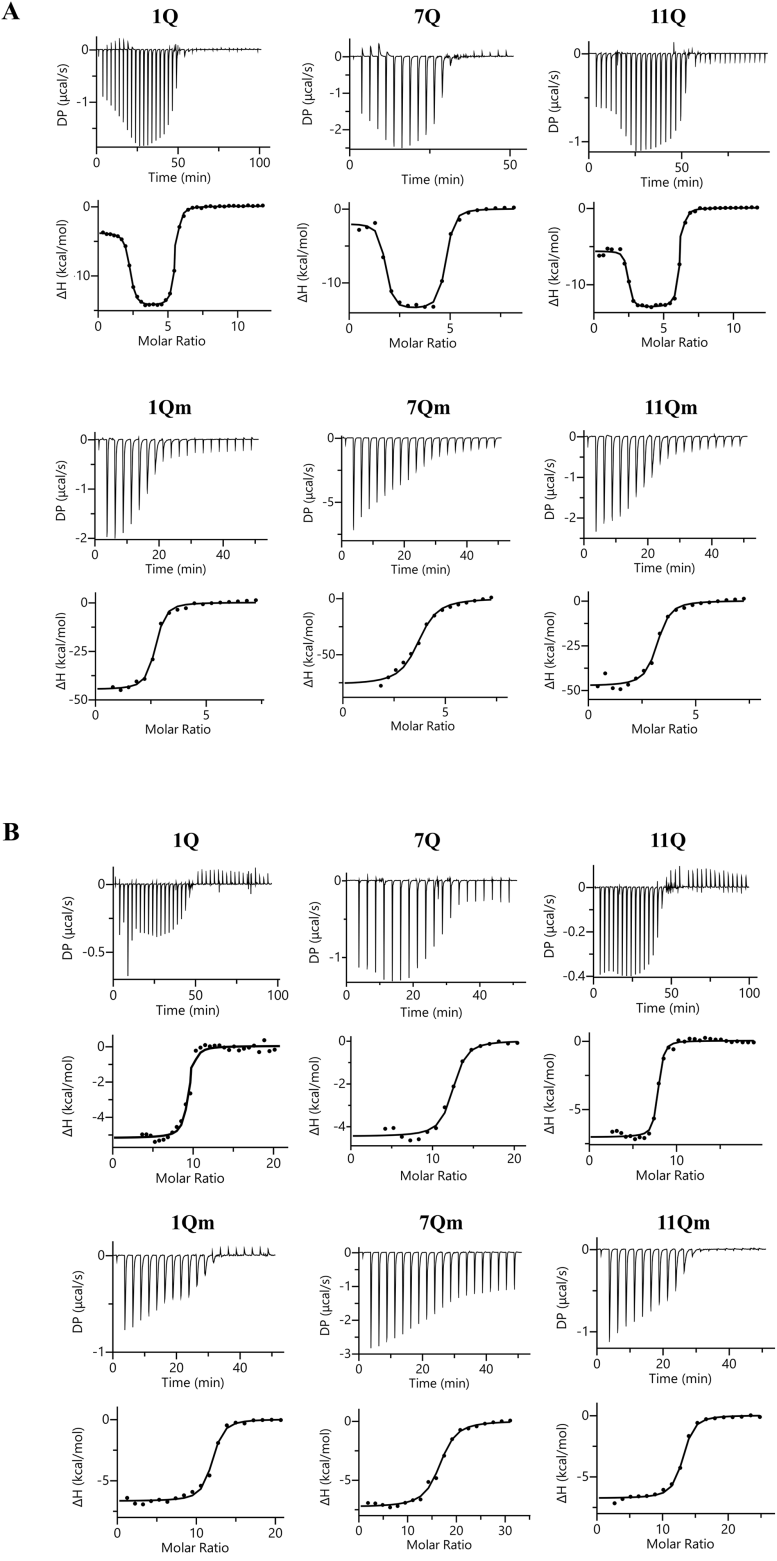
Representative raw data (upper panels) and integration of the peaks (bottom panels) with the best fit of the ‘One Set of Sites’ or ‘Two Sets of Sites’ model (only in the case of wild-type sequences and TMPyP4 ligand). The data were obtained after titrations of 1Q, 7Q, and 11Q G-quadruplexes and 1Qm, 7Qm, and 11Qm mutants with TMPyP4 (A) or BRACO-19 (B) ligands.

Among all studied RNA G-quadruplexes (1Q, 7Q, and 11Q), we could distinguish two sets of binding sites in the interaction with the TMPyP4 ligand. At the first set, the stoichiometry of TMPyP4 ligand binding could be approximated to ~ 3 (1Q and 11Q) or ~4 (7Q), while the second set of binding sites for the TMPyP4 with a stoichiometry of ~ 2 (Fig 4A, Table 2). TMPyP4 ligand binding is characterized by similar (within the margin of SD) parameters and mode, regardless of the G-quadruplex sequence; however, the 11Q demonstrates the highest affinity to the TMPyP4 (Table 2). The first type of binding site exhibits lower affinity to TMPyP4 with K_D_ in the mid-nanomolar range (~100−250 nM, Table 2), where the binding of the ligand is driven entirely by the enthalpy. The second type of TMPyP4 binding sites binds the ligand with several hundred-fold higher affinity, and this interaction was mainly driven by the entropy changes (Table 2). For the BRACO-19 ligand, the binding curves exhibited one significant inflection. Since the first inflection is inconspicuous, the model assuming the existence of one type of equivalent binding site was fitted to the obtained data (Fig 4B). Fitting titration curves of 1Q and 11Q resulted in one set of binding sites with similar K_D_ values of ~162 and 106 nM, respectively, and stoichiometry of ~8 and ~9, respectively (Table 2). In the case of 7Q, the interaction with BRACO-19 seemed to be more than twice as weak as the K_D_ is ~ 530 nM (Table 2). Interactions of all RNA G-quadruplexes with BRACO-19 were driven by enthalpy, although the entropy contribution was still significant (Table 2). The overall stoichiometry of BRACO-19 binding was almost twice as high as that of the TMPyP4 ligand, which could be explained by the fact that BRACO-19 molecular mass is more than 2-fold less than the molecular mass of TMPyP4 (Table 2).

**Table 2:** The binding parameters from the calorimetric titration of the wild-type and mutated variants of 1Q, 7Q, and 11Q with TMPyP4 and BRACO-19 ligands.

	Parameters	1Q	1Qm	7Q	7Qm	11Q	11Qm
**TMPyP4**	**N**_**1**_	3.3 ± 0.1	2.6 ± 0.1	4.4 ± 2.0	3.7 ± 0.2	3.1 ± 0.2	3.02 ± 0.02
	**K**_**d1**_ **[nM]**	258 ± 84	146 ± 48	276 ± 42	699 ± 144	106 ± 2	217 ± 82
	**ΔH**_**1**_ **[kcal/mol]**	−13.0 ± 2.2	−42.1 ± 3.8	−12.8 ± 1.3	−77.0 ± 4.3	−12.7 ± 0.6	−46.7 ± 1.4
	**-TΔS**_**1**_ **[kcal/mol]**	4.0 ± 1.9	32.8 ± 4.0	3.8 ± 1.4	70.0 ± 2.3	3.2 ± 0.6	37.6 ± 1.6
	**N**_**2**_	1.9 ± 0.4	–	2.5 ± 1.2	–	1.9 ± 0.5	–
		**K**_**d2**_ **[nM]**	2.8 ± 2.3		1.2 ± 0.9		0.3 ± 0.1
		**ΔH**_**2**_ **[kcal/mol]**	−2.6 ± 1.6		−2.1 ± 0.2		−4.3 ± 1.9
		**-TΔS**_**2**_ **[kcal/mol]**	−9.1 ± 1.0		−10.2 ± 0.3		−8.6 ± 1.7
**BRACO-19**	**N**	8.5 ± 1.0	12.0 ± 0.7	10.2 ± 2.6	16.2 ± 0.4	8.7 ± 1.6	12.8 ± 0.4
	**K**_**d**_ **[nM]**	162 ± 5	540 ± 213	530 ± 33	1580 ± 160	106 ± 8	584 ± 30
	**ΔH [kcal/mol]**	−4.8 ± 0.6	−6.8 ± 0.2	−4.4 ± 0.1	−7.4 ± 0.1	−6.2 ± 1.1	−6.7 ± 0.4
	**-TΔS** **[kcal/mol]**	−4.5 ± 0.6	−1.8 ± 0.4	−4.2 ± 0.1	−0.5 ± 0.1	−3.3 ± 1.1	−1.8 ± 0.4

The G-to-A mutations introduced to the G-quadruplex sequences affect the binding affinity and stoichiometry of the studied ligands. In the case of the TMPyP4 compound, the stoichiometry was reduced to ~3 (1Qm and 11Qm) or ~4 (7Qm), and this effect is limited to the low-affinity set of binding sites. Based on the analysis of the binding curves (Fig 4A), the first inflation completely disappears. We can explain this phenomenon by the fact that the mutation introduction may disrupt the folded RNA G-quadruplex structure, resulting in a loss of some additional TMPyP4 binding clefts. The affinity of TMPyP4 binding to the “low-affinity” set of binding sites was not affected significantly by the introduced mutations in the case of 1Q and 11Q, whereas in the case of 7Q, it was reduced by half. It is noteworthy that the binding of TMPyP4 to all mutated sequences caused large enthalpy and entropy changes, possibly due to the structurization of the unstructured sequences in the presence of a large TMPyP4 porphyrin ring. Interestingly, the described mutations also reduced the affinity for BRACO-19 by threefold (in 1Qm and 7Qm) and by fivefold (in 11Qm), while simultaneously increasing the binding stoichiometry (Fig 3B, Table 2). Moreover, these new stoichiometry values seem to be directly proportional to the length of sequences, as the N of binding is ~ 12, ~ 16, and ~13 for 1Qm, 7Qm, and 11Qm, respectively, whereas the length of sequences is 25, 32, and 28 nucleotides, respectively. This phenomenon can indicate that about two nucleotides could create the binding site for one BRACO-19 molecule in mutated sequences. The BRACO-19 binding curves for the mutated sequences are also devoid of the first vague inflection, which was observed for wild-type sequences. Mutations in G-rich regions also caused an increase in the enthalpy contribution to the binding and lowered the entropy contribution. Moreover, Libera et al. described the molecular mechanism of the interaction between BRACO-19 and human telomeric G-quadruplex [25]. The authors reported that modifying the topology of G4 may contribute to ligand interactions and binding modes [25].

In general, we noticed the differences in the binding properties between TMPyP4 and BRACO-19 compounds toward wild-type and mutated RNA G4s. It can be concluded that the cationic porphyrin is characterized by a higher binding affinity compared to the acridine derivative. This finding may result from different interaction mechanisms for these ligands, which remain unclear.

### 3.3 UV melting measurements of the IAV RNA G-quadruplexes

In general, UV spectroscopy is used to study the thermal stability of DNA and RNA structures, including G-quadruplexes, by monitoring changes in their absorbance as a function of temperature. Typically, the G4 structures exhibit a characteristic hypochromic effect at ~ around 295 nm due to the base stacking in the G-tetrads. To gain insight into the thermal stability of the IAV RNA G4s upon ligand binding, we carried out UV melting measurements. Although we performed measurements at two wavelengths: 260 and 295 nm, we chose to analyze the melting profile at 295 nm.

**Fig 5:**
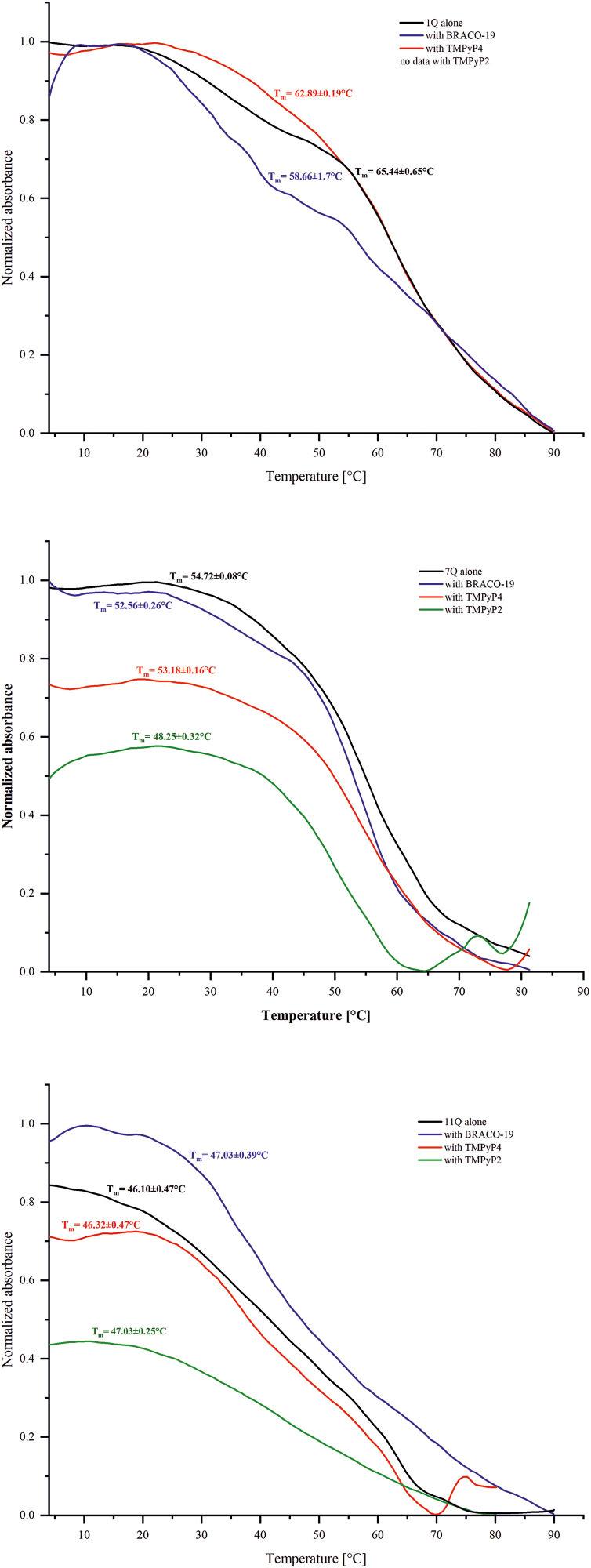
Representative UV melting curves of RNA oligomers in the presence or absence of ligands with T_m_ values obtained by monitoring the melting profile at 295 nm. The T_m_ values were obtained as the average of three replicates.

As a result, we were unable to obtain reliable thermodynamic parameters using this method; therefore, we focused on the melting profiles and changes in the melting temperature determined for 1Q, 7Q, and 11Q alone and with the addition of ligands (TMPyP4, BRACO-19, and TMPyP2). The obtained results showed that the melting curves of RNA oligomers (1Q, 7Q, and 11Q), both in the absence and presence of ligands, were inverted (Fig 5), indicating the formation of G-quadruplexes. This observation suggests that the ligand binding does not disrupt the folding of G4 structures under the experimental conditions. Furthermore, based on the UV melting analysis, we determined the melting temperature values (T_m_) for all RNA oligomers (without and with ligand addition), as shown in Fig 5.

Our results revealed only slight differences in the thermal stability of the 1Q, 7Q, and 11Q variants upon the ligand addition (Fig 5). Minor destabilization of the G-quadruplex structure was observed for 1Q in the presence of the TMPyP4 compound. The T_m_ values of 1Q and 1Q with TMPyP4 were 65.4 and 62.9 °C, respectively (Fig 5). A larger destabilization was noticed in the case of 1Q with BRACO-19, where the presence of this ligand caused a decrease in T_m_ by 6.8 °C (Figure X). For 1Q with the TMPyP2 compound, reliable results could not be obtained. According to our UV melting analysis for 7Q, the TMPyP4 presence induced slight changes in thermal stability (7Q alone T_m _= 54.7 °C vs. 7Q with TMPyP4 T_m_ = 55.5 °C, Fig 5). A subtle difference was revealed for 7Q after the addition of BRACO-19, where this compound induced a destabilization effect, decreasing its melting temperature by 2.2 °C. Surprisingly, the highest decrease in T_m_ value was observed in the case of 7Q with TMPyP2, by 6.5 °C, as shown in Fig 5. By analyzing the results obtained for 11Q, we found only minor differences between thermal stability after the addition of the tested ligands. More specifically, the exact change in melting temperature of 11Q was noticed in the presence of BRACO-19 and TMPyP2 compounds; the increase by 0.9 °C was recorded (Fig 5). In the case of 11Q with TMPyP4 ligand, the difference between ligand-bound and ligand-free conditions was negligible (only 0.2 °C in T_m_ values). We conclude that the thermal stability of 11Q was only minimally affected by these ligands. Moreover, based on our observations, we suggest that TMPyP4, BRACO-19, and TMPyP2 compounds can influence the melting temperature of RNA G4 structures, but only within a limited range under the conditions tested.

### 3.4 Circular dichroism measurements of the IAV RNA G-quadruplexes upon ligand titration

Circular dichroism (CD) spectroscopy provides information on the geometry of nucleic acid structures and various types of tertiary interactions. It is a simple and relatively fast technique for evaluating the G4 folding topology and assessing its structural features. The unique CD spectral signatures help monitor conformational changes in G4s under experimental conditions. Overall, CD spectroscopy is commonly used to determine the topology of G4s, cation effect, G4/ligand interactions, and ligand-induced stabilization, being a low-resolution complement to high-resolution techniques [5,10,11,18]. Therefore, we applied CD spectroscopy as a complementary technique to check the effect of TMPyP4 and BRACO-19 ligands on the RNA G4 structure.

**Fig 6:**
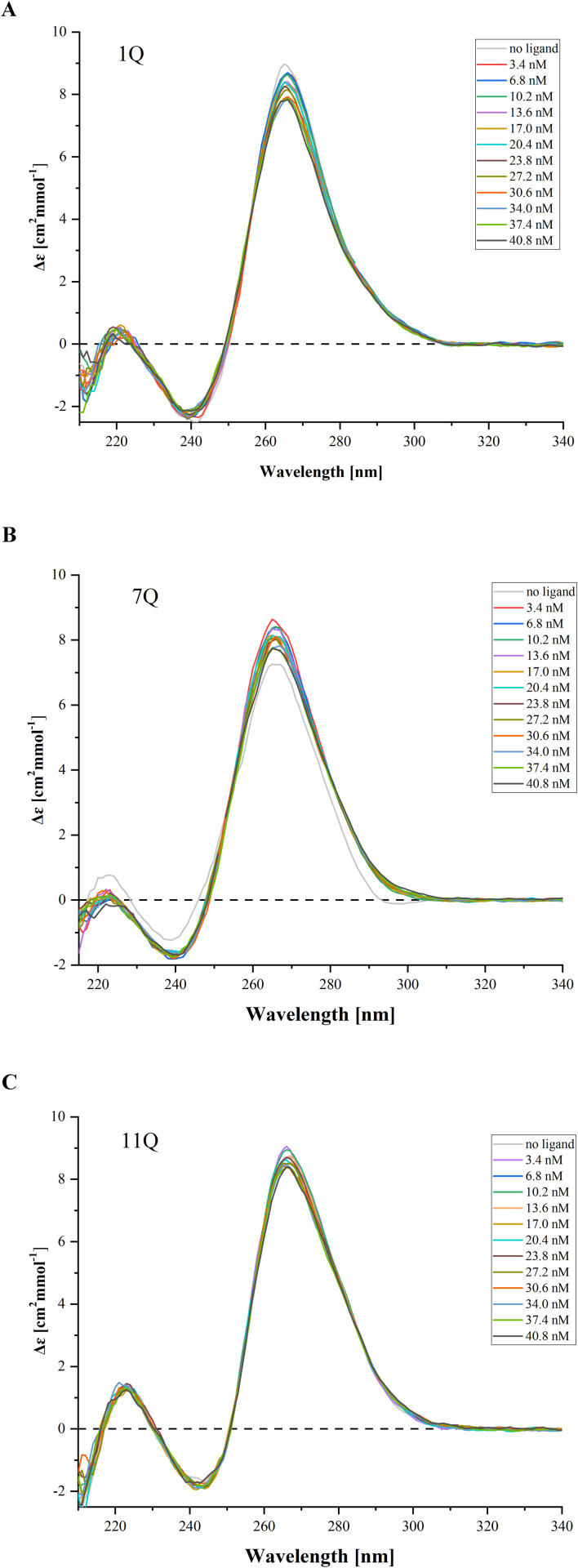
Circular dichroism spectra for pre-folded RNA G4s oligomers: 1Q (A), 7Q (B), and 11Q (C) with TMPyP4 increasing concentrations.

**Fig 7:**
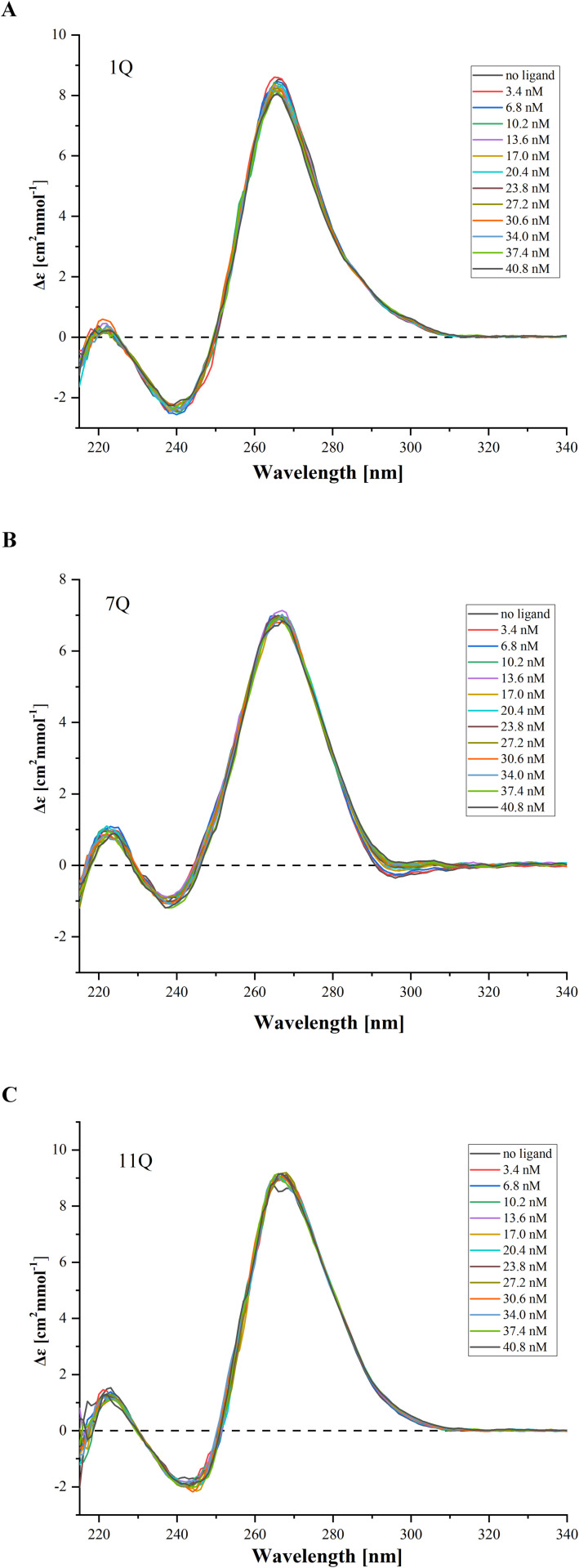
Circular dichroism spectra for pre-folded RNA G4s oligomers: 1Q (A), 7Q (B), and 11Q (C) with BRACO-19 increasing concentrations.

The CD analysis was conducted for three RNA G4s with increasing concentrations of TMPyP4 and BRACO-19 compounds. As a result, the CD spectra of 1Q, 7Q, and 11Q are presented in Figs 6 and 7. They are dominated by a strong positive band near 265 nm and a smaller negative band at 240 nm, typical for the parallel G-quadruplex structure. In general, the cationic porphyrin under tested conditions did not affect the positions of peaks, indicating that the 1Q, 7Q, and 11Q structures retained the parallel topology (Fig 6). A similar trend was noticed upon the addition of the BRACO-19 compound (Fig 7).

Importantly, we found minimal changes in the CD spectrum intensity that reflect the increase in the concentration of both ligands. However, no global RNA G-quadruplex structure differences were detected (Figs 4 and 5). We found that the G4-specific ligands in the solution slightly reduced the intensity of the CD spectra at 265 nm during the titration for 1Q, 7Q, and 11Q variants. The abovementioned findings suggest that the TMPyP4 and BRACO-19 compounds could not disturb the RNA G-quadruplex structure under experimental conditions.

### 3.5 Circular dichroism measurements of the RNA sequences from the PR8 genome

**Fig 8:**
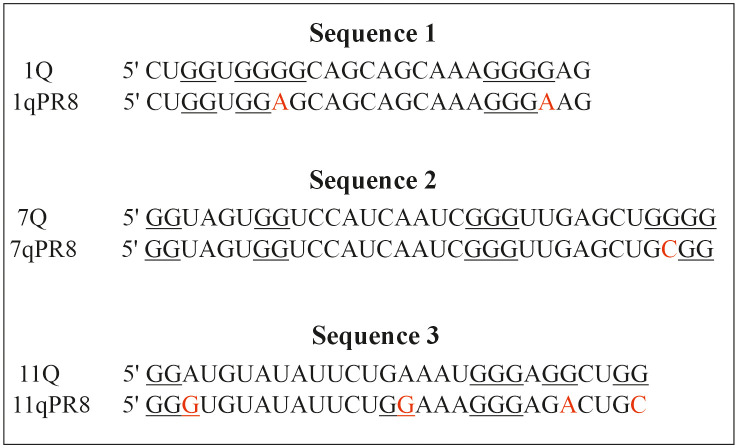
The differences in nucleotide composition of G-rich sequences from the Cal2009 and PR8 strains used in our study. Red color indicates the point mutations; the G-tracts are underlined.

The CD spectroscopy was used here to evaluate the structural conformations of RNA sequences from the A/Puerto Rico/8/34 (PR8) genome. We aimed to investigate whether differences in nucleotide composition of G-rich motifs influence the geometry of RNA. Fig 8 presents three G-rich sequences from the IAV Cal2009 (1Q, 7Q, and 11Q) and PR8 (1qPR8, 7qPR8, and 11qPR8) genomes with marked mutations in guanine tracts.

**Fig 9:**
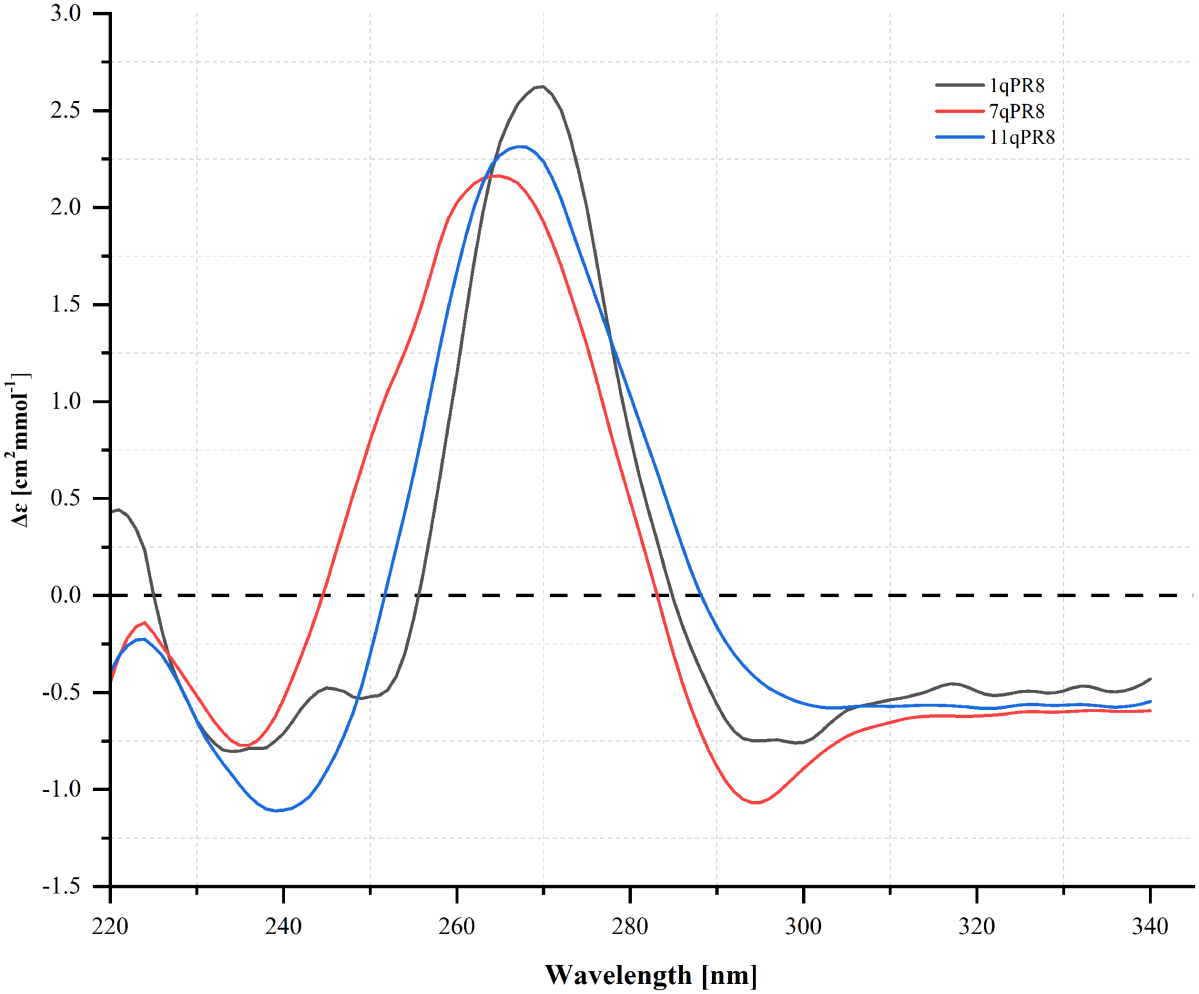
Circular dichroism spectra of 1qPR8, 7qPR8, and 11qPR8 oligomers.

We obtained the CD profiles for three RNA oligomers, named 1qPR8, 7qPR8, and 11qPR8, which are shown in Fig 9. In general, the resultant CD profiles for these RNAs have similar features. Namely, the CD spectrum of 1qPR8 displays two negative bands near 235 and 295 nm, and a single maximum at 270 nm (Fig 9). For 7qPR8, we observed a broad positive band between 245 and 285 nm, with a maximum near 265 nm (Fig 9). Additionally, two minima were also found near 235 and 295 nm (7qPR8, Fig 9). In the case of 11qPR8, a single positive peak from 250 to 290 nm with a maximum at 268 nm was noticed (Fig 9). We also observed two negative bands, one with a maximum near 240 nm and the second near 295 nm (Fig 9). It has been reported in the literature that the typical CD profile of parallel G-quadruplex displays a strong, positive band near 265 nm and a negative peak around 245 nm [26–28].

According to our CD analysis, we suggest that 1qPR8, 7qPR8, and 11qPR8 can fold into G4 structures. However, we noted a slight deviation from the expected spectral features. Specifically, in the resulting CD spectra, a negative peak near 295 nm appeared (Fig 9). This result may indicate the existence of higher-order G4 structures or an equilibrium between different conformations, such as A-form RNA or hairpin. Additionally, this finding highlights the structural heterogeneity and conformational flexibility of the analyzed RNAs. Furthermore, these effects can be due to various factors, not only the nucleotide composition of the G-rich sequence but also the RNA concentration used in the CD experiment, or the presence of metal cations in the solution.

Interestingly, we used RNAStructure 6.1 Software for RNA secondary structure prediction for sequences from both Cal2009 (1Q, 7Q, and 11Q) and PR8 (1qPR8, 7qPR8, and 11qPR8) strains. All predicted RNA structures are shown in S4 Fig in the Supplementary Materials. The differences observed in RNA folding patterns confirm the structural diversity among the tested sequences. It can be concluded that the nucleotide composition of G-rich sequences influences the G-quadruplex folding behaviour.

### 3.6 Fluorescence spectroscopy analysis of the IAV RNA G-quadruplex-ligand interactions

**Fig 10:**
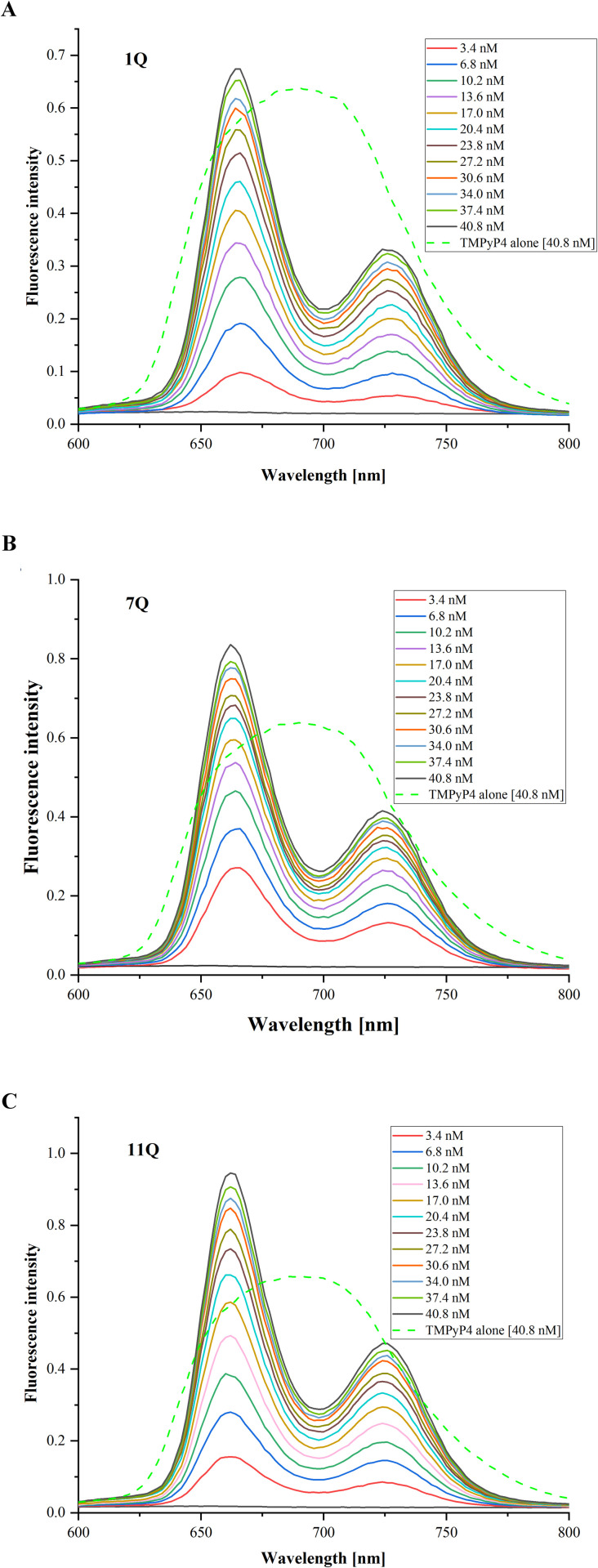
The fluorescence titration spectra of the TMPyP4 ligand in the absence and presence of pre-folded RNA G4s oligomers: 1Q (A), 7Q (B), and 11Q (C). The spectrum of TMPyP4 alone in the buffer is indicated as a light green dashed line.

To gain further insight into the interaction between RNA G4s and selected ligands, we applied fluorescence spectroscopy. We expected changes in the fluorescence spectrum and intensity when a selected ligand binds to a G-quadruplex structure. Herein, the fluorescence titration spectra of pre-folded 1Q, 7Q, and 11Q G4s (keeping RNA concentration constant) with increasing concentrations of TMPyP4 and BRACO-19 ligands were recorded. The fluorescence emission spectra for the TMPyP4 compound, measured at wavelengths ranging from 600 to 800 nm with excitation at 433 nm, are presented in Fig 10. Overall, the measurements were continued until the changes in TMPyP4 fluorescence intensity became negligible. We observed a decrease in the distance between peaks at emission maxima with increasing concentrations of TMPyP4 up to six-fold excess to the RNA G4s. Consequently, the fluorescence spectrum of TMPyP4 alone features a single broad emission peak centered near 700 nm (Fig 10, light green dashed line). In contrast, the fluorescence spectra changed after the addition of RNA G4 oligomers, and two emission maxima at 660 nm and near 730 nm appeared (Fig 10). The same trend was observed in all cases (1Q, 7Q, and 11Q), which suggests the TMPyP4 compound interacts with the G-quadruplexes present in the solution. In other words, different shapes of recorded spectra confirm that RNA G4s can interact with the cationic porphyrin under experimental conditions.

**Fig 11:**
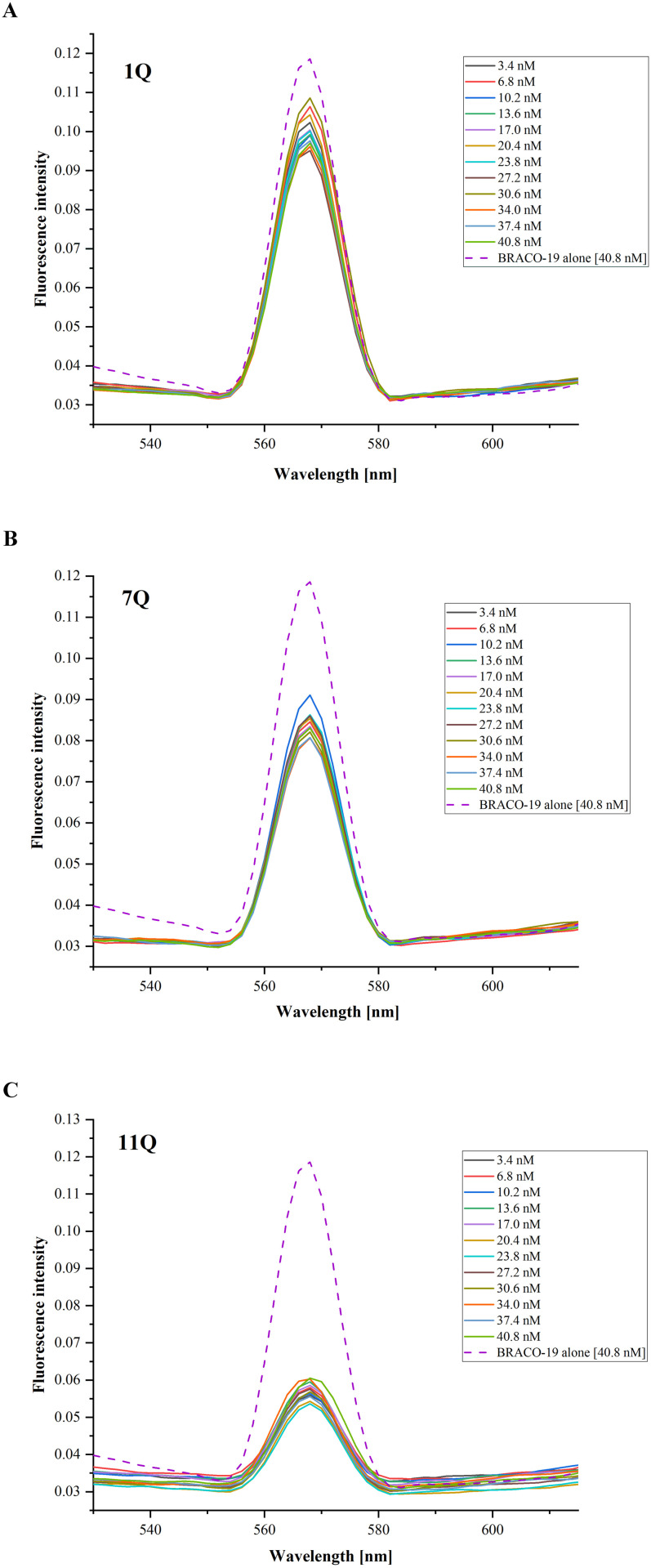
The fluorescence titration spectra of the BRACO-19 ligand in the absence and presence of pre-folded RNA G4s oligomers: 1Q (A), 7Q (B), and 11Q (C). The spectrum of BRACO-19 alone in the buffer is indicated as a purple dashed line.

As mentioned earlier, we also investigated the effects of the three G4s on the BRACO-19 fluorescence spectrum. The fluorescence spectra of the BRACO-19 compound with 1Q, 7Q, and 11Q G4s were recorded in the range from 500 to 650 nm with excitation set at 285 nm. Similar to the TMPyP4 ligand, the measurements were conducted until the changes in BRACO-19 fluorescence intensity were insignificantly small. The results for all RNA G4s variants with increasing concentrations of acridine compound are presented in Fig 11. We observed a different trend for BRACO-19 upon the addition of 1Q, 7Q, and 11Q G4s compared to the TMPyP4 ligand. The overall shape of the recorded spectra remained unchanged when RNA G4s were present in the solution. In all cases, the spectral profile of BRACO-19 fluorescence is characterized by a single positive peak with a maximum near 570 nm (Fig 11). However, comparing the fluorescence intensity of the BRACO-19 ligand *vs.* the addition of G-quadruplexes, one can notice its decrease after the addition of RNA G4s (Fig 11). Surprisingly, the most significant change in fluorescence intensity was found in the case of the 11Q variant (BRACO-19 alone *vs.* 11Q with 40.8 nM BRACO-19, Fig 11C). In contrast, the smallest was for the 1Q variant (BRACO-19 alone versus 1Q with 40.8 nM BRACO-19, Fig 11A). Additionally, the changes in fluorescence intensity across varying concentrations of BRACO-19 were relatively small (Fig 11). This observation may suggest that the presence of RNA G4s in the solution did not significantly affect the fluorescence of the acridine derivative.

### 3.7 Modeling of 1Q G-quadruplex

**Fig 12:**
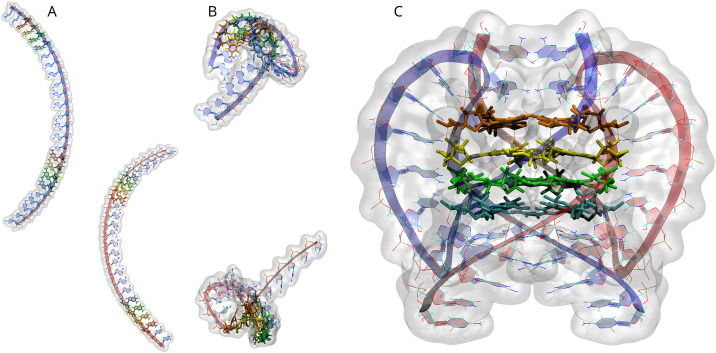
Three main stages of building the 1Q model. A) Generating RNA strands; B) Intramolecular folding; C) Intermolecular folding. The RNA molecules are shown using the new ribbon representation and highlighted with a white, semi-transparent surface. The two strands forming the G-quadruplex are coloured blue and red. The four layers of tetrads are shown in liquorice representation and coloured red, yellow, green, and blue.

To supplement our biophysical and experimental approaches, we applied molecular modeling to simulate the folding behaviour of 1Q G-quadruplex, providing further insights into its structural stability and topology. Given the 1Q RNA sequence, the program generated its two identical elongated RNA strands with stacking base interactions positioned according to imposed distance restraints to rigidify the initial structure (Fig 12A). Subsequently, guanine (G) quartets, expected to form adjacent pairs via intramolecular Hoogsteen hydrogen bonding, were defined. Following π–π stabilization of the four adjacent stacking guanines by additional distance restraints, RNA molecules were folded according to distance restraints between guanines involved in the intramolecular Hoogsteen interactions, resulting in the formation of two halves of a four-layered bimolecular G-quadruplex (Fig 12B). Next, these folded RNA molecules were brought together using an additional set of distance restraints between guanines involved in the intermolecular Hoogsteen interactions to form a complete, bimolecular, four-tetrad G-quadruplex model (Fig 12C).

**Fig 13:**
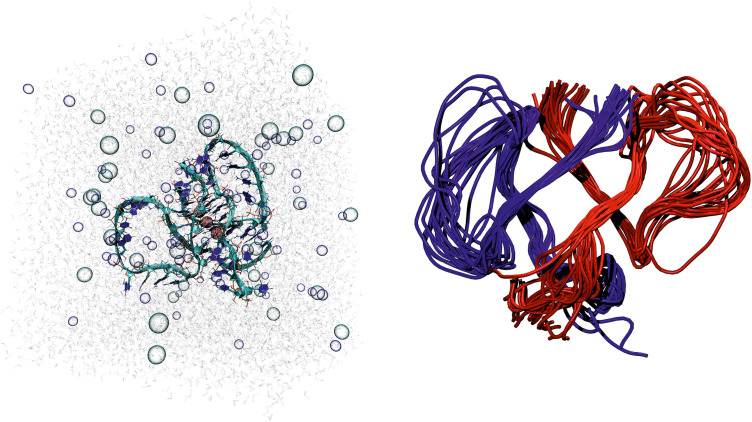
Simulation box with 1Q bimolecular G-quadruplex in the new ribbon representation, explicit water solution with ions (represented as transparent bubbles) during molecular dynamics simulation. The pink spheres are potassium ions in the channel of the core between the stacked G-tetrads (left panel). Representative structures of the 1Q G-quadruplex obtained during 300 ns of molecular dynamics simulations, superimposed using the phosphate groups of the core (consisting of 16 guanines) as the fitting centres. The two strands forming the G-quadruplex are shown in a tube representation of the RNA backbone and are colored blue and red. The core of the molecule remains stable, while the loops do not reach equilibrium during the entire simulation (right panel).

Subsequently, three potassium ions were inserted between the stacked guanine tetrads to stabilize the structure. The system was then placed in a simulation box filled with an explicitly represented 150 mM aqueous NaCl solution (Fig 13). The prepared G-quadruplex was subjected to a multiple-step minimization and equilibration protocol designed to gradually remove the imposed restraints. Initially, only the solvent atoms were relaxed, followed by a stepwise loosening of the G-quadruplex loops. In the final stage, all restraints, including those on the core formed by the four guanine tetrads, were fully released, and the system was further optimized. Due to the strong restraints required during G-quadruplex folding, the preparation process for molecular dynamics simulations, aiming to replicate physiological conditions, involved a gradual reduction of restraint strength by approximately 35-fold concurrently with a gradual temperature increase from 0 to 310 K in the constant volume conditions (NVT). The resulting G-quadruplex was then subjected to multi-stage molecular dynamics simulations in constant pressure (NPT). Representative G-quadruplex geometries obtained every 10 ps from approximately 300 ns of the production runs of molecular dynamics simulation were clustered into 22 distinct groups, as shown in the right panel of Fig 13.

Molecular dynamic simulations indicate that the bimolecular G-quadruplex remains stable, i.e., its four stacked tetrads persistently maintain their structure under physiological conditions, whereas the long loops do not reach equilibrium, as throughout the simulation, no loop adopts the same conformation twice. The results presented in the right panel of Fig 13 suggest that although 1Q RNA strands can form a G-quadruplex that remains stable under physiological conditions, their excessive flexibility, due to the dynamic behaviour of the long loops, may cause significant challenges for crystallization or NMR analysis of this bimolecular RNA system.

### 3.8 Biological studies of the influence of G4-specific ligand on the IAV minireplicon system activity

**Fig 14:**
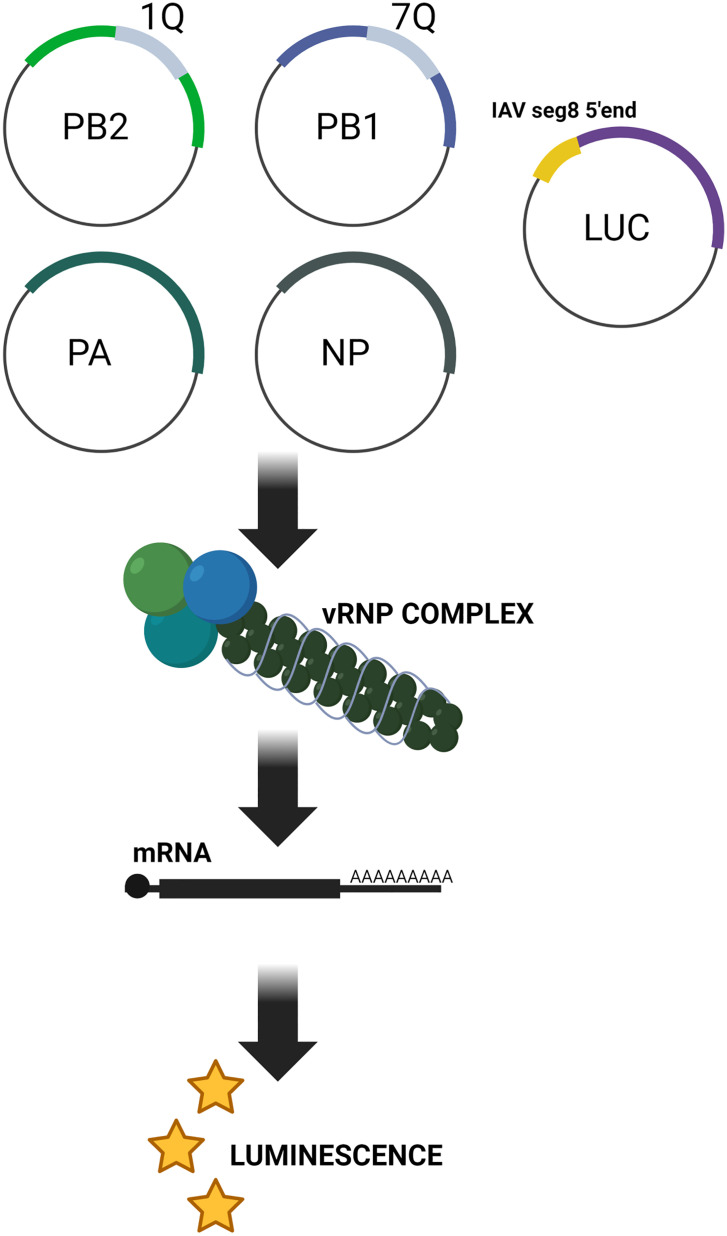
Scheme of the IAV minireplicon system used in this study.

Our previous studies have shown that identified G-rich motifs (1Q, 7Q, and 11Q) are located within segments encoding polymerase complex proteins (PB1 and PB2) and within the hemagglutinin (HA) segment [11]. Considering the localization of G4s within the vRNA segments, it can be assumed that these structures may play essential roles in regulating different steps of the viral life cycle. Recently, a non-infectious life cycle modeling system has been developed using reverse genetic methods [29,30]. This minireplicon system (also known as a minigenome) has been used in studies concerning the chemical targeting of a G-quadruplex RNA in the Ebola virus L gene [9]. Therefore, in our research, we aimed to examine the influence of G4-specific ligand binding on luciferase expression using the genomic vRNA analog system (Fig 14). This system relies on a viral mini-genome, which controls the expression of firefly luciferase (Luc). Within this minireplicon system, the expression of the viral proteins (PB2, PB1, PA, and NP) and vRNA-like transcript results in the *in vitro* reconstitution of active vRNP complexes. Those vRNP complexes generate the corresponding mRNA, which controls the expression of firefly luciferase with chemiluminescence activity. The luciferase is encoded under the influenza A virus segment 8 5´end promoter in the plasmid. Thus, its expression is only possible if the vRNP complex is reconstituted correctly.

A clear in our study, we used the IAV minireplicon system, which enables an accurate and rapid test of the activity of the vRNP complex based on its ability to replicate a virus-like RNA encoding the luciferase protein. Moreover, we assumed that adding the TMPyP4 ligand should, to some extent, inhibit minireplicon activity in the cells. Additionally, we decided to use another ligand in biological experiments, the analog TMPyP2, which is reported to have a lower affinity for the G-quadruplex and is therefore often used as a negative control compound in TMPyP4 activity studies [18–21]. Both compounds were confirmed not to have a toxic effect on the selected cell line using a cytotoxicity assay. The results of cytotoxicity of TMPyP4 and TMPyP2 compounds in HEK 293T cell culture are presented in S2 Table in Supplementary Materials.

Notably, based on our previous studies, we found the difference in nucleotide composition of G-rich sequences (1Q and 7Q) between A/California/04/2009 (Cal2009) and A/Puerto Rico/8/34 (PR8) genomes, as indicated earlier in Fig 8 (*3.5 Circular dichroism measurements of the RNA sequences from the PR8 genome section*). We reported that PQS regions from segments 1 and 2 of PR8 differ from the corresponding PQS regions in the Cal2009 genome. The 1Q sequence from PR8 contains A residues instead of G residues within two different G-tracts in the Cal2009 genome. In contrast, the 7Q sequence from PR8 has a C residue instead of a G residue in the G-rich region, compared with the sequence from Cal2009. From this observation, it may be suggested that differences in nucleotide composition can affect the conformational changes of G-quadruplex structures in the PR8 strain genome. This finding motivated us to use the PR8 minireplicon system in our investigation.

**Fig 15:**
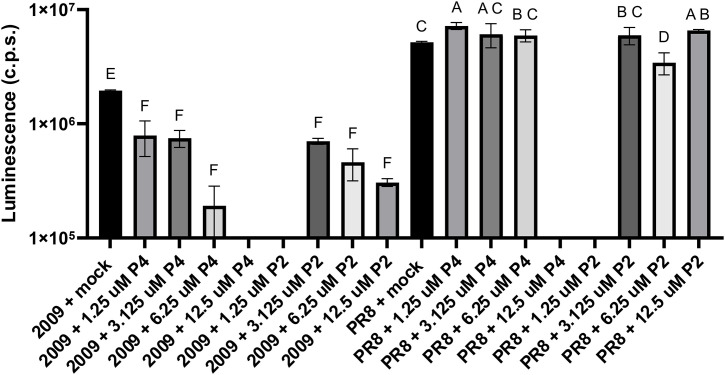
The inhibition of Cal2009 and PR8 minireplicon activity in HEK 293T cells after TMPyP4 or TMPyP2 treatment. All data are presented as the means from two biological replicates, each subjected to three technical replicates. Error bars reflect the SD.

By analyzing the luciferase luminescence signals, we conclude that both ligands markedly inhibited minireplicon activity compared to the negative control (TMPyP4 IC_50 _= 4.04 µM; TMPyP2 IC_50 _= 4.32 µM). Additionally, the observed effect appears to be dose-independent, as all selected ligand concentrations caused minireplicon activity inhibition (Fig 15). During the experimental optimization, we decided to select three concentrations for each ligand. We agreed not to use the lowest TMPyP2 concentration (1.25 µM) as no ligand effect was observed after its addition. Conversely, the effect on the minireplicon system after the addition of the highest TMPyP4 concentration (12.5 µM) did not differ from the effect caused by half of that concentration. Moreover, our findings suggest a strain-dependent inhibitory effect of the ligands. In the case of PR8 minireplicon, ligands do not show such a strong repressive effect compared to the negative control, but instead seem to have no significant influence on minireplicon activity (Fig 15). Interestingly, TMPyP2 at a concentration of 6.25 µM has an inhibitory effect on PR8 minireplicon system activity (Fig 15). On the other hand, the TMPyP4 ligand at the lowest concentration appears to have a stimulatory effect on the PR8 minireplicon, as its addition increased the luminescence signal. At this moment, the mechanism of this stimulation is unknown, but we suspect that it may be a nonspecific effect. Further structural and bioinformatic investigations are necessary, starting with RNA sequence analysis, examining its interaction with the ligand, and exploring the possible function of RNA G4s. The fact that we observe an inhibitory effect only in the case of the Cal2009 minireplicon highlights the importance of our selected PQS motifs that were identified in the vRNA of IAV A/California/04/2009 strain and confirmed to contain mutations within the IAV A/Puerto Rico/8/34 vRNA. It is also worth noting that these two minireplicon systems have different activity levels. Even though we used three times higher plasmid concentrations for the Cal2009 minireplicon, the activity of the PR8 minireplicon remained noticeably higher.

Although we assessed binding affinity using the ITC method and examined the effect of ligands on thermal stability and folding of RNA G4s, a clear correlation with the observed inhibitory effects in the cellular environment was not consistently evident across the tested ligands. The relationship between G4 thermal stability, folding dynamics, and ligand accessibility is complex and may not be directly correlated. The minireplicon assay revealed a significant inhibition of IAV replication. However, the structural features of RNA G4/ligand interactions observed in our biophysical studies do not necessarily predict the ligand’s effect in a cellular context, such as in the minireplicon system. Several factors may explain this lack of direct correlation. For example, the complex and dynamic nature of RNA G4s in a cellular environment, where folding kinetics, RNA–protein interactions, and structural accessibility can significantly influence ligand binding and functional outcomes. Additionally, ligand specificity and potential off-target effects should also be considered. G4-specific ligands may bind to other RNA or DNA structures or even interact with proteins, affecting replication independently of G4 binding. Therefore, effects observed on minireplicon activity may not strictly reflect G4 stabilization.

## 4 Discussion

The influenza virus, which causes the common flu, has a high epidemic and pandemic potential, resulting from its high genome variability. It is known that viral biological processes, such as replication and pathogenicity, are controlled by the secondary and tertiary structures of viral RNA. RNA is utilized throughout the replication cycle, highlighting its fundamental role in IAV biology.

The secondary structure of vRNA, including IAV vRNA, is highly conserved among different strains, suggesting its importance for the viral life cycle. Additionally, it has been shown that RNA structural motifs, such as G-quadruplexes, are present within the viral genomes and can have important biological functions [1,31–33]. Therefore, we recommend further research into the correlation between vRNA structure and function. In our previous paper, we identified and described structural aspects of G-quadruplexes from the IAV genome [11].

A significant number of G4-specific ligands have been studied, some of which show potential as antiviral therapeutic agents [34,35]. For example, TMPyP4 and BRACO-19 compounds stabilize G-quadruplex formation and could be inhibitors of viral replication [10,18]. Given the findings that the presence of G-quadruplexes can induce reverse transcription pausing, [12,13] here, we tested both TMPyP4 and BRACO-19 ligands on the RNA G4s from the IAV genome. However, due to the known lack of site specificity of these ligands, we primarily treated them as indicators of the presence and functional relevance of G-quadruplexes in the IAV replication, suggesting that these sequences may serve as targets for anti-viral drug development.

In our current study, we first examined the effect of TMPyP4 and BRACO-19 ligands on the inhibition of cDNA synthesis. The obtained results revealed some differences between the reverse transcription pausing after the addition of both tested compounds. The changes in the intensity patterns for 1q, 7q, and 11q on the gels may result from the different stability of the RNA G-quadruplexes. The relatively weak binding or effect observed in the RT stop assay suggests that viral RNA G4s may have structural features that hinder effective ligand recognition or stabilization, which represents an important finding. Additionally, our UV melting experiments support this, showing that the thermal stability (T_m_) of the RNA G-quadruplexes shows only minor changes with ligand addition, with some cases showing a slight decrease, and others minimal increases. These subtle shifts indicate that, under our experimental conditions, the ligands do not strongly stabilize the RNA G4s. Notably, previous studies on TMPyP4 and BRACO-19 ligands in the context of polymerase pausing have yielded varying results. This reflects differences in G4 folding topology, sequence context, or assay conditions. For example, Chashchina et al. observed that TMPyP4 and BRACO-19 induced minimal or no detectable polymerase pausing in native promoter sequences of selected oncogenes [36]. They noted the pause sites induced by BRACO-19 in the *MYC* promoter at high ligand concentrations. Based on this result, the authors suggest that the tested compounds exhibit low selectivity for G4 structures or provide insignificant stabilization of G4s [36]. Another example is the study by Zhou and co-workers, who investigated a small molecule targeting the RNA G-quadruplex structure in a negative-sense RNA virus [9]. The authors showed that the TMPyP4 inhibits RNA synthesis at the G4 site. The RNA synthesis pausing was gradually promoted at G4 sites upon treatment with increasing concentrations of the TMPyP4 in the presence of potassium cations [9]. Moreover, Richter and co-workers showed that the TMPyP4 ligand interacts with Herpes Simplex Virus-1 G4s and inhibits polymerase progression *in vitro* [18]. A strong pausing effect was observed, and with the increasing concentrations of TMPyP4, one/two additional bands at the stop site suggested an increased stabilization of G4 structure. In contrast, the TMPyP2 compound did not show any effects different from the untreated oligonucleotide [18].

Furthermore, a combination of the ITC technique, CD measurements, UV melting, and fluorescence spectroscopy allowed us to characterize the RNA G-quadruplex-ligand interactions. Our biophysical analyses provided information about the binding properties of TMPyP4 and BRACO-19 ligands to three RNA G4s from the IAV genome. They revealed some interaction changes that may be caused by different binding mechanisms of the ligand. The structural features of the ligand and its target (in this case, G-quadruplexes) can explain the changes in the affinity and specificity among TMPyP4 and BRACO-19 compounds binding to the same RNA G4s. Several investigations concerning the characterization of the ligand-G4 interactions were reported [37–39]. For instance, Zhang and co-workers analyzed the binding ability of ligands, including BRACO-19 and TMPyP4, to G4s from the enterovirus A71 (EV-A71) genome [37]. The authors found that G4-specific ligands stabilize EV-A71 G-quadruplex structures with high affinity. However, they observed different affinity and selectivity of such interactions [37]. Our results are consistent with literature reports, suggesting that various ligands and their structural rigidity can affect the behavior of G-quadruplex structures.

The main difference between TMPyP4-G-quadruplex and BRACO-19-G-quadruplex interactions lies in their binding modes. TMPyP4 binds externally, mainly through stacking on base pairs and loop regions, and avoids direct stacking on the central G-quartet. For instance, crystallographic studies by Neidle and co-workers showed that intercalation is clearly not the mode of interaction of TMPyP4 with the G4 structures [40]. Notably, Han *et al.* reported that TMPyP4 appears to bind to parallel G4s through external stacking at the ends rather than through intercalation between the G-tetrads [20]. The authors concluded that intercalative binding, although less favored than external binding, may occur, though it remains undetectable using the employed methods, such as the photocleavage assay [20].

In contrast, BRACO-19 binds via an intercalative mode, sandwiched between G-quartets, and also interacts with the grooves through its side chains. This binding pattern makes BRACO-19 a more specific and selective ligand for the G-quadruplex structure [41]. Recently, Si *et al.* reported experimental and molecular docking results showing that BRACO-19 binds with the residues of the DNA G4. The π -moiety of BRACO-19 interacts with the G-tetrad through π-π stacking and the side chain interaction with the groove of DNA G4 [42].

Furthermore, the folding topology and molecularity of 1Q, 7Q, and 11Q structures may also influence their interaction with G4-specific compounds. Based on our CD analysis, we identified the parallel-stranded topology for these three G4s (1Q, 7Q, and 11Q). However, G-quadruplexes as polymorphic structures can differ in their loop regions, the number of stacking guanines within the strand, and their arrangements. Although the binding mechanisms of TMPyP4 and BRACO-19 ligands to RNA G4s are still unclear, results from multiple biophysical methods presented above suggest that these compounds can influence the 1Q, 7Q, and 11Q G-quadruplex structure formation. Taken together, our data emphasize that the interactions between selected ligands and viral RNA G4s may be more complex and less predictable than previously thought, highlighting the need for case-by-case structural and functional validation.

Recent studies have explored G-quadruplex binders as potential antiviral agents, e.g., a paper relating to G-quadruplex as a therapeutic target was described by Lv and colleagues in 2022 [5]. The authors confirmed G4 structure formation within the chikungunya virus (CHIKV) genome using different biophysical techniques. Importantly, they determined the inhibition of CHIKV genome replication by BRACO-19 and TMPyP4 ligands. They also demonstrated that the production of infectious virus was inhibited by these compounds [5]. Our previous studies showed that 1Q and 7Q sequences are present in the segments encoding polymerase complex proteins, whereas 11Q is found within the hemagglutinin (HA) segment [11]. Considering the above findings, we examined the effect of TMPyP4 and TMPyP2 compounds on the IAV minireplicon system activity. The non-infectious life cycle modeling system has been developed using reverse genetic methods [29,30]. It was previously employed, for example, in the chemical targeting of a G4 RNA in the Ebola virus L gene [9].

Here, we assumed that G-quadruplex structures could play an important role in the regulation of the viral replication cycle; therefore, we used the minireplicon system. Importantly, the IAV A/California/04/2009 strain causes dangerous pandemic outbreaks in contrast to the IAV A/Puerto Rico/8/34, a lab-adapted strain, which serves as a model for seasonal influenza viruses. An important point is that we found a difference in the nucleotide composition of G-rich sequences between these two strains within the G-quadruplex-forming regions. Therefore, the use of the minireplicon system based on Cal2009 and PR8 for *in vitro* studies is justified. We found that the compounds we utilized affect the viral polymerase activity in the IAV minireplicon reporter assay, suggesting that they can interact with the G-quadruplexes formed within the vRNA of polymerase complex protein segments.

Additionally, we observed differences in IAV replication inhibition among IAV strains, with the inhibitory effect being more significant for the Cal2009 strain. Therefore, whether this naturally occurring subtle difference, specifically the single nucleotide substitution in the G-rich region within the IAV genome, can be related to viral pathogenicity remains an open question. We hypothesize that this single nucleotide substitution in the PR8 genome could affect G-quadruplex formation, leading to different gene regulation patterns. The G4-specific ligand is not an efficient inhibitor of IAV replication, as the compound has no binding target.

In summary, our paper demonstrates the molecular and structural basis of how TMPyP4 and BRACO-19 bind to selected IAV RNA G4s from the A/California/04/2009 strain. This is the first study to indicate that these structures are formed within this pandemic influenza A virus strain and can interact with G4-specific ligands. Our investigation has proven that both compounds can interact with RNA G-quadruplexes under experimental conditions; however, their binding modes differ. Moreover, the findings reported here show that TMPyP4 and BRACO-19 inhibit reverse transcription reactions *in vitro*. Finally, we demonstrated that TMPyP4 effectively inhibits IAV minireplicon activity in a cellular environment. Collectively, our study suggests that targeting viral RNA G4s by chemical compounds might contribute to the mechanism of their biological activity. Therefore, by employing chemical ligands, we have shown that the PQS regions within the IAV genome represent potential target sites for the development of new, sequence-specific therapeutics. Moreover, we suggest that RNA G4s may play a potentially important role in viral replication, a finding that requires further detailed biological study. Overall, the viral RNA G-quadruplexes could be novel targets for developing an anti-influenza strategy.

## Supporting information

S1 Table List of RNA and DNA oligomers used in this work. (DOCX)

S2 Table Cytotoxicity of TMPyP4 and TMPyP2 compounds in HEK 293T cell culture. (DOCX)

S1 Fig The full gel images of the inhibition of cDNA synthesis catalyzed by the reverse transcriptase in the presence of TMPyP4 ligand. (TIF)

S2 Fig The full gel images of the inhibition of cDNA synthesis catalyzed by the reverse transcriptase in the presence of BRACO-19 ligand. (TIF)

S3 Fig The resultant gels from the cDNA synthesis catalyzed by the reverse transcriptase in the presence of TMPyP2 for 1q/1qm, 7q/7qm, and 11q/11qm variants. (TIF)

S4 Fig RNA secondary structures for sequences from both Cal2009 (1Q, 7Q, and 11Q) and PR8 (1qPR8, 7qPR8, and 11qPR8) strains. RNA secondary structures were predicted and generated using RNAStructure 6.1 software.
(TIF)
