# Correction: The interaction of RNA G-quadruplexes from the influenza A virus vRNA with TMPyP4 and BRACO-19 ligands

**DOI:** 10.1371/journal.pone.0356274

**Published:** 2026-08-17

**Authors:** The PLOS One Staff

In Table 2, some of the parameters obtained from the ITC measurements were incorrectly positioned and are associated with the wrong RNA oligomers and ligands. Please see the correct Table 2 below.

The publisher apologizes for the errors.

**Table 2:** The binding parameters from the calorimetric titration of the wild-type and mutated variants of 1Q, 7Q, and 11Q with TMPyP4 and BRACO-19 ligands.

	Parameters	1Q	1Qm	7Q	7Qm	11Q	11Qm
**TMPyP4**	**N_1_**	3.3 ± 0.1	2.6 ± 0.1	4.4 ± 2.0	3.7 ± 0.2	3.1 ± 0.2	3.02 ± 0.02
	**K_d1_ [nM]**	258 ± 84	146 ± 48	276 ± 42	699 ± 144	106 ± 2	217 ± 82
	**ΔH_1_ [kcal/mol]**	-13.0 ± 2.2	-42.1 ± 3.8	-12.8 ± 1.3	-77.0 ± 4.3	-12.7 ± 0.6	-46.7 ± 1.4
	**-TΔS_1_ [kcal/mol]**	4.0 ± 1.9	32.8 ± 4.0	3.8 ± 1.4	70.0 ± 2.3	3.2 ± 0.6	37.6 ± 1.6
	**N_2_**	1.9 ± 0.4	–	2.5 ± 1.2	–	1.9 ± 0.5	–
	**K_d2_ [nM]**	2.8 ± 2.3		1.2 ± 0.9		0.3 ± 0.1	
	**ΔH_2_ [kcal/mol]**	-2.6 ± 1.6		-2.1 ± 0.2		-4.3 ± 1.9	
	**-TΔS_2_ [kcal/mol]**	-9.1 ± 1.0		-10.2 ± 0.3		-8.6 ± 1.7	
**BRACO-19**	**N**	8.5 ± 1.0	12.0 ± 0.7	10.2 ± 2.6	16.2 ± 0.4	8.7 ± 1.6	12.8 ± 0.4
	**K_d_ [nM]**	162 ± 5	540 ± 213	530 ± 33	1580 ± 160	106 ± 8	584 ± 30
	**ΔH [kcal/mol]**	-4.8 ± 0.6	-6.8 ± 0.2	-4.4 ± 0.1	-7.4 ± 0.1	-6.2 ± 1.1	-6.7 ± 0.4
	**-TΔS [kcal/mol]**	-4.5 ± 0.6	-1.8 ± 0.4	-4.2 ± 0.1	-0.5 ± 0.1	-3.3 ± 1.1	-1.8 ± 0.4
