# Supplementary material for: The interaction of RNA G-quadruplexes from the influenza A virus vRNA with TMPyP4 and BRACO-19 ligands
Source: PLoS One. 2025 Nov 19;20(11):e0335975. doi: 10.1371/journal.pone.0335975 (PMC12629423; doi:10.1371/journal.pone.0335975)
Supplement: S1 Table — (DOCX) [file pone.0335975.s001.docx]

**Table S1.** List of RNA and DNA oligomers used in this work.

|  | | **Name** | **Sequence (5′-3′)** | **Application** |
| --- | --- | --- | --- | --- |
| **RNA** | **wild-type** | 1q | CUGGUGGGGCAGCAGCAAAGGGGAGCCGCUUGUACCGA | RT stop assay |
|  |  | 7q | GGUAGUGGUCCAUCAAUCGGGUUGAGCUGGGG CCGCUUGUACCGA |  |
|  |  | 11q | GGAUGUAUAUUCUGAAAUGGGAGGCUGGCCGCUUGUACCGA |  |
|  | **mutant** | 1qm | CUGGUGGAGCAGCAGCAAAAGGAAGCCGCUUGUACCGA |  |
|  |  | 7qm | GGUAGUGGUCCAUCAAUCGGAUUGAGCUGAGG CCGCUUGUACCGA |  |
|  |  | 11qm | GGAUGUAUAUUCUGAAAUGGAAGACUUGCCGCUUGUACCGA |  |
| **DNA** |  | primer 1 | FAM -TCGGTACAAGCGG |  |
| **RNA** |  | 1Q | CUGGUGGGGCAGCAGCAAAGGGGAG | ITC method |
|  |  | 7Q | GGUAGUGGUCCAUCAAUCGGGUUGAGCUGGGG |  |
|  |  | 11Q | GGAUGUAUAUUCUGAAAUGGGAGGCUGG |  |
|  |  | 1Qm | CUGGUGGAGCAGCAGCAAAAGGAAG |  |
|  |  | 7Qm | GGUAGUGGUCCAUCAAUCGGAUUGAGCUGAGG |  |
|  |  | 11Qm | GGAUGUAUAUUCUGAAAUGGAAGACUUG |  |
| **DNA** |  | For-PB1 insert | ACCATGGATGTCAATCCGACTCTAC | minireplicon system preparation |
|  |  | Rev-PB1 insert | CAATGGTGGAACAGATCTTCATGATCTC |  |
|  |  | For-PB1 vector | GAAGATCTGTTCCACCATTGAAGAACTCAG |  |
|  |  | Rev-PB1 vector | GTCGGATTGACATCCATGGTGGTAC |  |
|  |  | For-PB2 insert | CACCATGGAGAGAATAAAAGAACTGAGAGA |  |
|  |  | Rev-PB2 insert | GCTCGAGCTAATTGATGGCCATCCGAA |  |
|  |  | For-PB2 vector | GGCCATCAATTAGCTCGAGCTAGCAGAT |  |
|  |  | Rev-PB2 vector | CTTTTATTCTCTCCATGGTGATGGGTACCATGCA |  |
|  |  | For-PA insert | GGTACCACCATGGAAGACTTTGTGCGAC |  |
|  |  | Rev-PA insert | CTAGCTCGAGCTACTTCAGTGCATGTGTGAG |  |
|  |  | For-PA vector | ACTGAAGTAGCTCGAGCTAGCAGATCTTTTT |  |
|  |  | Rev-PA vector | AAGTCTTCCATGGTGGTACCATGCATCGATG |  |
|  |  | For-NP insert | CATGGTACCACCATGGCGTCTCAAGGCA |  |
|  |  | Rev-NP insert | CTAGCTCGAGTCAACTGTCATACTCCTCTGCA |  |
|  |  | For-NP vector | TGACAGTTGACTCGAGCTAGCAGATCTTTTTCC |  |
|  |  | Rev-NP vector | GACGCCATGGTGGTACCATGCATCGATG |  |
| Red color indicates point mutations in G-rich region. FAM – fluorescein modification at the 5′-end | | | | |
