# Supplementary material for: The interaction of RNA G-quadruplexes from the influenza A virus vRNA with TMPyP4 and BRACO-19 ligands
Source: PLoS One. 2025 Nov 19;20(11):e0335975. doi: 10.1371/journal.pone.0335975 (PMC12629423; doi:10.1371/journal.pone.0335975)
Supplement: S2 Table — (DOCX) [file pone.0335975.s002.docx]

**Table S2.** Cytotoxicity of TMPyP4 and TMPyP2 compounds in HEK 293T cell culture.

| **Compound concentration [μM]** | **Cellular viability** (% of cells alive within 48 h) | **Standard deviation** |
| --- | --- | --- |
| **TMPyP4** |  |  |
| 1.25 | 104.54 | ± 6.8 |
| 3.125 | 95.8 | ±6.86 |
| 6.25 | 89.01 | ±4.05 |
| 7.5 | 83.24 | ± 4.61 |
| **TMPyP2** |  |  |
| 3.125 | 98.50 | ± 5.25 |
| 6.25 | 89.68 | ± 6.64 |
| 12.5 | 78.38 | ± 4.64 |
