# Supplementary figures and images for: The interaction of RNA G-quadruplexes from the influenza A virus vRNA with TMPyP4 and BRACO-19 ligands
Source: PLoS One. 2025 Nov 19;20(11):e0335975. doi: 10.1371/journal.pone.0335975 (PMC12629423; doi:10.1371/journal.pone.0335975)

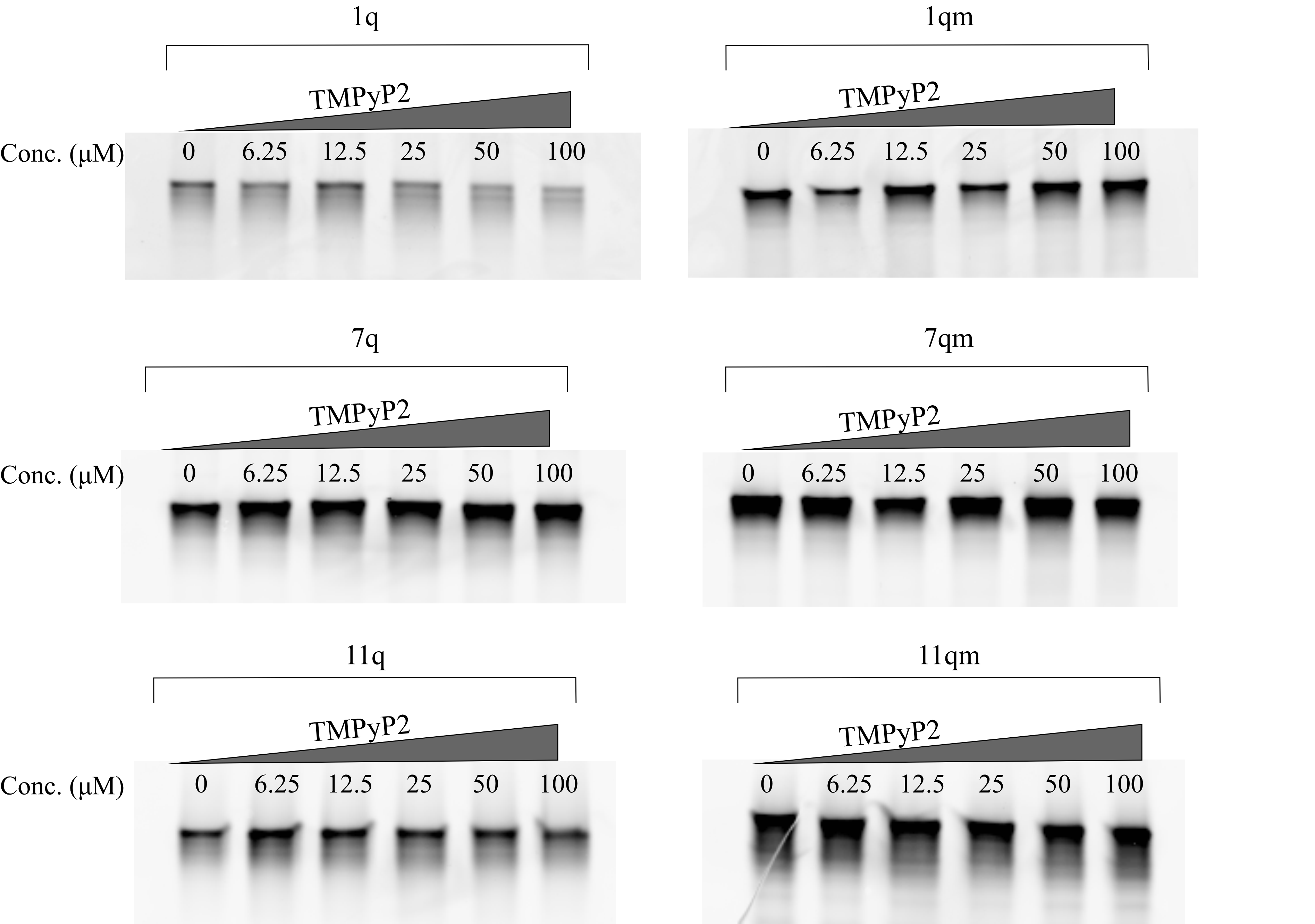

Supplement: S3 Fig — (TIF) [file pone.0335975.s005.tif]
